# Supplementary material for: Analysis of compositions of microbiomes with bias correction
Source: Nat Commun. 2020 Jul 14;11:3514. doi: 10.1038/s41467-020-17041-7 (PMC7360769; doi:10.1038/s41467-020-17041-7)
Supplement: Supplementary file 2 — Supplementary Information [file 41467_2020_17041_MOESM2_ESM.pdf]

Supplementary Information

Analysis of Compositions of Microbiomes with Bias Correction (ANCOM-BC)

Lin et al.

Supplementary Discussion

Inflated false positive rates of some standard methods

Two potential reasons why some differential abundance (DA) analysis methods for microbiome data result in inflated positive rates, and hence inflated false discovery rates (FDR), are as follows:

(1) The test statistic may not be designed for testing the hypothesis of interest. For example, the test statistic may be designed for testing hypothesis regarding relative abundance but is used for testing absolute abundance.

(2) Data are not properly normalized to account for bias due to variability in sampling fractions.

In the following we discuss some commonly used methods in the literature, namely, Wilcoxon rank sum test (with and without TSS)[1], DESeq2 [2], edgeR [3], metagenomeSeq [4]. We begin with the following simple lemma.

Lemma 0.1. *Suppose, for a taxon i, $E(\hat{\beta}_i) = \beta_i + \delta_i$, and $\widehat{SE}(\hat{\beta}_i)$ is $O_p(n^{-1/2})$. Further assume that, under H_0, $\beta_i = 0$, $\delta_i \neq 0$ and*

$$T_{\beta_i} = \frac{\hat{\beta}_i - \beta_i}{\widehat{SE}(\hat{\beta}_i)} \rightarrow_d N(\frac{\delta_i}{SE(\hat{\beta}_i)}, 1).$$

Suppose $z_{1-\alpha/2}$ is $(1-\alpha/2) \times 100$ percentile of standard normal distribution then the probability of Type I error associated with the critical region:

$$|T_{\beta_i = 0}| \geq z_{1-\alpha/2}$$

increases with sample size. Equivalently, the p-value based on $|T_{\beta_i=0}|$ stochastically decreases with n.

Proof. Note that under the null hypothesis we have T_{β_i} is centered at δ_i. Since $\delta_i \neq 0$, and $\widehat{SE}(\hat{\beta}_i)$ grows at the rate of $\sqrt{n}$, therefore $|T_{\beta_i}|$ stochastically increases with n, and p-value decreases stochastically. This results in inflated Type I error. □

In the following sections, suppose taxon i is not differentially abundant between two ecosystems or two groups. For simplicity of exposition, we assume the sample sizes are equal between the two groups.

Wilcoxon rank-sum test with no normalization

Suppose for $k = 1, \ldots, n, O_{i1k} \sim_{iid} F_{i1}$ and $O_{i2k} \sim_{iid} F_{i2}$. Under no normalization, the Wilcoxon rank-sum test aim to test the following hypotheses

$$H_0 : F_{i1} = F_{i2}$$
$$H_1 : F_{i1} \neq F_{i2}$$

The test statistic is given by:

$$U = \frac{1}{n^2} \sum_{k=1}^{n} \sum_{k'=1}^{n} I(O_{i1k} \leq O_{i2k'}). \tag{1}$$

Asymptotically, under the null hypothesis we know that:

$$U \sim AN(\frac{1}{2}, \frac{1}{6n}). \tag{2}$$

The basic assumption made by the above U statistic is that under the null hypothesis O_{i1k} is equally likely to be small or large compared to O_{i2k}. Thus the indicator random variable $I(O_{i1k} \leq O_{i2k})$ has the same distribution as $I(O_{i2k} \leq O_{i1k})$. Note that in the existing implementation of these tests the samples are not normalized for unequal sampling fractions. Therefore under the null hypothesis, the U statistic is not centered at $\frac{1}{2}$. Hence the Type I error is not controlled at α according to Lemma 0.1.

Wilcoxon rank-sum test with TSS

TSS normalization transforms the absolute abundance table into the relative abundance table. Using these relative abundance data, for $k = 1, \ldots, n, r_{i1k} \sim_{iid} G_{i1}$ and $r_{i2k} \sim_{iid} G_{i2}$, the Wilcoxon Rank-Sum test is used for testing the following hypotheses:

$$H_0 : G_{i1} = G_{i2}$$
$$H_1 : G_{i1} \neq G_{i2}.$$

Under the above normalization, even if the expected absolute abundance of a taxon is same between two ecosystems, its relative abundances may not be same. Thus, testing the null hypothesis of equality of relative abundance of a taxon between two ecosystems is not equivalent to the null hypothesis that the absolute abundances are equal. Furthermore, the Wilcoxon rank-sum test applied directly to the relative abundance data ignores the compositional structure. Consequently, asymptotically the Type I error will not be controlled as indicated in Lemma 0.1.

DESeq2

DESeq2 assumes a negative-binomial model for absolute abundances. Thus, the observed count data and the corresponding parameters are modeled as follows:

$$\begin{aligned}
&O_{ijk} \sim NB(s_{jk}q_{ij}, \phi_i) \\
&s_{jk} = \underset{i:O_i^R \neq 0}{\text{median}} \frac{O_{ijk}}{O_i^R} \\
&\log q_{ij} = \beta_{i0} + \beta_{i1} I(j = 1), \quad j = 1, 2 \\
&\hat{\beta}_{i1} = \arg\max_{\beta_{i1}} (\sum_{j=1}^{2} \sum_{k=1}^{n} \log f_{NB}(O_{ijk}; s_{jk}q_{ij}, \phi_i) + \Lambda(\beta))
\end{aligned} \tag{3}$$

where

(1) $O_i^R = (\prod_{j=1}^{2} \prod_{k=1}^{n} O_{ijk})^{\frac{1}{2n}}$,

(2) $\Lambda(\beta) = -(\frac{\beta_{i0}^2}{2\sigma_0^2} + \frac{\beta_{i1}^2}{2\sigma_1^2})$,

(3) σ_0^2, σ_1^2 are prior variances for β_{i0}, β_{i1}, respectively.

DESeq2 first scales the OTU table by the normalization factor s_{jk}, and then tests for differential abundance, consequently it does not take into account the uncertainty associated with s_{jk}.

Recall from the regression framework of ANCOM-BC that:

$$\begin{aligned} E(O_{ijk}) &= c_{jk}\theta_{ij} \\ E(y_{ijk}) &= d_{jk} + \mu_{ij} \end{aligned} \tag{4}$$

Compared to (3), DESeq2 estimates the sampling fraction c_{jk} by s_{jk}, i.e. $\hat{c}_{jk}^{\text{MED}} = s_{jk}$ and therefore $\hat{d}_{jk}^{\text{MED}} = \log s_{jk}$. Thus, we have

$$\begin{aligned} \hat{d}_{jk}^{\text{MED}} &= \underset{i:O_i^R \neq 0}{\text{median}}(\log O_{ijk} - \frac{1}{2n}\sum_{j=1}^{2}\sum_{k=1}^{n}\log O_{ijk}) \\ &= \underset{i:O_i^R \neq 0}{\text{median}}(y_{ijk} - \frac{1}{2n}\sum_{j=1}^{2}\sum_{k=1}^{n} y_{ijk}) \\ &= \underset{i:O_i^R \neq 0}{\text{median}}(d_{jk} + \mu_{ij} + \epsilon_{ijk} - \frac{1}{2n}\sum_{j=1}^{2}\sum_{k=1}^{n} y_{ijk}) \\ &= \underset{i:O_i^R \neq 0}{\text{median}}(d_{jk} - \bar{d}_{\cdot\cdot} + \mu_{ij} - \bar{\mu}_{i\cdot} + \epsilon_{ijk} - \bar{\epsilon}_{i\cdot\cdot}) \\ &= d_{jk} - \bar{d}_{\cdot\cdot} + \underset{i:O_i^R \neq 0}{\text{median}}(\mu_{ij} - \bar{\mu}_{i\cdot} + \epsilon_{ijk} - \bar{\epsilon}_{i\cdot\cdot}) \\ &:= d_{jk} - \bar{d}_{\cdot\cdot} + \mu_{a_{jk}j} - \bar{\mu}_{a_{jk}\cdot} + \epsilon_{a_{jk}jk} - \bar{\epsilon}_{a_{jk}\cdot\cdot}. \end{aligned} \tag{5}$$

In the expressions a_{jk} denotes the index that corresponds to the taxon for which $\underset{i:O_i^R \neq 0}{\text{median}}(\mu_{ij} - \bar{\mu}_{i\cdot} + \epsilon_{ijk} - \bar{\epsilon}_{i\cdot\cdot}) = \mu_{a_{jk}j} - \bar{\mu}_{a_{jk}\cdot} + \epsilon_{a_{jk}jk} - \bar{\epsilon}_{a_{jk}\cdot\cdot}$. Averaging over all samples $k = 1, 2, \ldots, n$ in group j, we get

$$\bar{\hat{d}}_{j\cdot}^{\text{MED}} = \bar{d}_{j\cdot} - \bar{d}_{\cdot\cdot} + \tilde{\mu}_{\cdot(j)j} - \tilde{\mu}_{\cdot(j)\cdot} + \tilde{\epsilon}_{\cdot(j)j\cdot} - \tilde{\epsilon}_{\cdot(j)\cdot\cdot}. \tag{6}$$

Since each subject k in group j, may potentially have a different taxon that yields the median value $\mu_{a_{jk}j} - \bar{\mu}_{a_{jk}\cdot} + \epsilon_{a_{jk}jk} - \bar{\epsilon}_{a_{jk}\cdot\cdot}$, in the above expression $\tilde{x}$ represents the mean of variable x taken over the suitable subset of taxa. Secondly, the notation $x_{\cdot(j)}$ represents the mean taken within group j.

The test statistic for DESeq2 is of the form:

$$W_i^{\text{DESeq2}} = \frac{\hat{\mu}_{i1} - \hat{\mu}_{i2} - \hat{\delta}^{\text{MED}}}{\text{SE}(\hat{\mu}_{i1} - \hat{\mu}_{i2} - \hat{\delta}^{\text{MED}})} \tag{7}$$

The MED estimator of the bias term is:

$$\begin{aligned} \hat{\delta}^{\text{MED}} &:= \bar{\hat{d}}_{1\cdot}^{\text{MED}} - \bar{\hat{d}}_{2\cdot}^{\text{MED}} \\ &= \bar{d}_{1\cdot} - \bar{d}_{2\cdot} + \{\tilde{\mu}_{\cdot(1)1} - \tilde{\mu}_{\cdot(1)\cdot} + \tilde{\epsilon}_{\cdot(1)1\cdot} - \tilde{\epsilon}_{\cdot(1)\cdot\cdot}\} - \{\tilde{\mu}_{\cdot(2)2} - \tilde{\mu}_{\cdot(2)\cdot} + \tilde{\epsilon}_{\cdot(2)2\cdot} - \tilde{\epsilon}_{\cdot(2)\cdot\cdot}\} \\ &= \delta + \{\tilde{\mu}_{\cdot(1)1} - \tilde{\mu}_{\cdot(1)\cdot} + \tilde{\epsilon}_{\cdot(1)1\cdot} - \tilde{\epsilon}_{\cdot(1)\cdot\cdot}\} - \{\tilde{\mu}_{\cdot(2)2\cdot} - \tilde{\mu}_{\cdot(2)\cdot} + \tilde{\epsilon}_{\cdot(2)2\cdot} - \tilde{\epsilon}_{\cdot(2)\cdot\cdot}\} \end{aligned} \tag{8}$$

Note that $E(\tilde{\epsilon}_{\cdot(j)j\cdot} - \tilde{\epsilon}_{\cdot(j)\cdot\cdot}) = 0$. However, unless $E_{\mathcal{S}}(\tilde{\mu}_{\cdot(1)1} - \tilde{\mu}_{\cdot(1)\cdot}) = 0$ and $E_{\mathcal{S}}(\tilde{\mu}_{\cdot(2)2} - \tilde{\mu}_{\cdot(2)\cdot}) = 0$, where the subscript $\mathcal{S}$ denotes the collection of all suitable subsets of taxa $\{1, 2, \ldots, m\}$, the MED estimator does not estimate the bias term in the null hypothesis unbiasedly, i.e.

$$E(\hat{\delta}^{\text{MED}}) \neq \delta. \tag{9}$$

Thus, under the null hypothesis

$$E(\hat{\mu}_{i1} - \hat{\mu}_{i2} - \hat{\delta}^{\text{MED}}) \neq 0 \tag{10}$$

As seen from the figures presented in the main text as well as this supplementary text, the normalization method used in DESeq2 works sometimes (Supplementary Fig. 2, 8), and sometimes fails to eliminate the bias due to variability in the sampling fraction (Fig. 3, Supplementary Fig. 1). Consequently, the test statistic used in DESeq2 intrinsically tests a biased hypothesis and hence from Lemma 0.1, it can potentially inflate the false positive rate.

edgeR

Similar to DESeq2, edgeR assumes a negative-binomial distribution for absolute abundance data:

$$\begin{aligned} &O_{ijk} \sim \text{NB}(O_{\cdot jk} s_{jk} p_{ij}, \phi_i) = \text{NB}(M_{jk} p_{ij}, \phi_i) \\ &\log p_{ij} = \beta_{i0} + \beta_{i1} I(j = 1), \quad j = 1, 2 \end{aligned} \tag{11}$$

where

(1) s_{jk} = normalization factor,

(2) M_{jk} = effective library size, which is the product of original library size and normalization factor,

(3) p_{ij} is the relative abundance of taxon j in experimental group j.

The upper-quartile (UQ) normalization used in edgeR is described as follows. Let

$$\begin{aligned} \hat{c}_{jk}^{\text{UQ}} &= s_{jk} = \underset{i:O_{ijk}>0}{\text{UQ}} (\frac{O_{ijk}}{O_{\cdot jk}}) \\ \hat{d}_{jk}^{\text{UQ}} &= \log \hat{c}_{jk}^{\text{UQ}}, \end{aligned} \tag{12}$$

where UQ(X) is the upper quartile of X. Then

$$\begin{aligned} \hat{d}_{jk}^{\text{UQ}} &= \underset{i:O_{ijk}>0}{\text{UQ}} (\log O_{ijk} - \log O_{\cdot jk}) \\ &\text{(Apply Taylor's expansion)} \\ &\approx \underset{i:O_{ijk}>0}{\text{UQ}} (y_{ijk} - \log c_{jk}\theta_{\cdot j} - \frac{1}{c_{jk}\theta_{\cdot j}}(O_{\cdot jk} - c_{jk}\theta_{\cdot j})) \\ &= \underset{i:O_{ijk}>0}{\text{UQ}} (d_{jk} + \mu_{ij} + \epsilon_{ijk} - d_{jk} - \log \theta_{\cdot j} - \frac{O_{\cdot jk}}{c_{jk}\theta_{\cdot j}} + 1) \\ &= 1 - \log \theta_{\cdot j} - \frac{O_{\cdot jk}}{c_{jk}\theta_{\cdot j}} + \underset{i:O_{ijk}>0}{\text{UQ}} (\mu_{ij} + \epsilon_{ijk}) \\ &:= 1 - \log \theta_{\cdot j} - \frac{O_{\cdot jk}}{c_{jk}\theta_{\cdot j}} + \mu_{a_{jk}j} + \epsilon_{a_{jk}jk} \end{aligned} \tag{13}$$

Similar to DESeq2, for the kth sample in the jth group, a_{jk} represents the index for the taxon such that $\underset{i:O_{ijk}>0}{\text{UQ}} (\mu_{ij} + \epsilon_{ijk}) = \mu_{a_{jk}j} + \epsilon_{a_{jk}jk}$.

Averaging over all sample $k = 1, 2, \dots n$, we get

$$\bar{\hat{d}}_{j\cdot}^{\text{UQ}} = 1 - \log \theta_{\cdot j} - \bar{x}_{j\cdot} + \tilde{\mu}_{\cdot(j)j} + \tilde{\epsilon}_{\cdot(j)j\cdot} \tag{14}$$

As noted earlier, since each subject k in group j, may potentially have a different taxon that yields the upper quartile value $\mu_{a_{jk}j} + \epsilon_{a_{jk}jk}$, in the above expression $\tilde{x}$ represents the mean of variable x taken over the suitable subset of taxa. Secondly, the notation $\cdot(j)$ represents the mean taken within group j. $\bar{x}_{j\cdot}$ is the average of $\frac{O_{\cdot jk}}{c_{jk}\theta_{\cdot j}}$ over group j.

Thus, the UQ estimator of the bias term in the null hypothesis is

$$\begin{aligned}\hat{\delta}^{\text{UQ}} &:= \bar{\hat{d}}_{1\cdot}^{\text{UQ}} - \bar{\hat{d}}_{2\cdot}^{\text{UQ}} \\ &= (\log \theta_{\cdot 2} - \log \theta_{\cdot 1}) + (\bar{x}_{2\cdot} - \bar{x}_{1\cdot}) + (\tilde{\mu}_{\cdot(1)1} - \tilde{\mu}_{\cdot(2)2}) + (\tilde{\epsilon}_{\cdot(1)1\cdot} - \tilde{\epsilon}_{\cdot(2)2\cdot})\end{aligned} \tag{15}$$

Note that $E(\tilde{\epsilon}_{\cdot(1)1\cdot} - \tilde{\epsilon}_{\cdot(2)2\cdot}) = 0$. However, it is clear that the UQ estimator does not estimate the bias term in the null hypothesis unbiasedly, i.e. $E(\hat{\delta}^{\text{UQ}}) \neq \delta = \bar{d}_{1\cdot} - \bar{d}_{2\cdot}$.

Thus the UQ normalization method does not eliminate (even asymptotically) the bias due to variability in the sampling fraction. Consequently, the test statistic intrinsically tests a biased hypothesis and hence from Lemma 0.1, it inflates the false positive rate.

Comparing the model used in edgeR (11) with regression framework of ANCOM-BC, we note that:

$$E(O_{ijk}) = M_{jk} p_{ij} \tag{16}$$

Therefore, it is more reasonable to define the estimated sampling fraction by the effective library size. For instance, the effective library size using UQ (ELib-UQ):

$$\begin{aligned}\hat{c}_{jk}^{\text{ELib-UQ}} &= M_{jk} = O_{\cdot jk} s_{jk} \\ \hat{d}_{jk}^{\text{ELib-UQ}} &= \log \hat{c}_{jk}^{\text{ELib-UQ}}\end{aligned} \tag{17}$$

Hence, we have:

$$\begin{aligned}\hat{d}_{jk}^{\text{ELib-UQ}} &= \underset{i:O_{ijk}>0}{\text{UQ}} (\log O_{ijk}) \\ &= \underset{i:O_{ijk}>0}{\text{UQ}} (y_{ijk}) \\ &= \underset{i:O_{ijk}>0}{\text{UQ}} (d_{jk} + \mu_{ij} + \epsilon_{ijk}) \\ &= d_{jk} + \mu_{a_{jk}j} + \epsilon_{a_{jk}jk}\end{aligned} \tag{18}$$

As before, for the kth sample in the jth group, a_{jk} represents the index for the taxon such that $\underset{i:O_{ijk}>0}{\text{UQ}} (d_{jk} + \mu_{ij} + \epsilon_{ijk}) = d_{jk} + \mu_{a_{jk}j} + \epsilon_{a_{jk}jk}$.

Averaging over all sample $k = 1, 2, \dots n$, we get

$$\bar{\hat{d}}_{j}^{\text{ELib-UQ}} = \bar{d}_{j\cdot} + \tilde{\mu}_{\cdot(j)j} + \tilde{\epsilon}_{\cdot(j)j\cdot} \tag{19}$$

Since each subject k in group j may potentially have a different taxon that yields the upper quartile $\mu_{a_{jk}j} + \epsilon_{a_{jk}jk}$, in the above expression $\tilde{x}$ represents the mean of variable x taken over the suitable subset of taxa. Secondly, the notation $\cdot(j)$ represents the mean taken within group j.

Thus, the ELib-UQ estimator of the bias term in the null hypothesis is:

$$\begin{aligned}\hat{\delta}^{\text{ELib-UQ}} &:= \bar{\hat{d}}_{1\cdot}^{\text{ELib-UQ}} - \bar{\hat{d}}_{2\cdot}^{\text{ELib-UQ}} \\ &= \bar{d}_{1\cdot} - \bar{d}_{2\cdot} + (\tilde{\mu}_{\cdot(1)1} - \tilde{\mu}_{\cdot(2)2}) + (\tilde{\epsilon}_{\cdot(1)1\cdot} - \tilde{\epsilon}_{\cdot(2)2\cdot}) \\ &= \delta + (\tilde{\mu}_{\cdot(1)1} - \tilde{\mu}_{\cdot(2)2}) + (\tilde{\epsilon}_{\cdot(1)1\cdot} - \tilde{\epsilon}_{\cdot(2)2\cdot})\end{aligned} \tag{20}$$

Note that $E(\tilde{\epsilon}_{\cdot(1)1\cdot} - \tilde{\epsilon}_{\cdot(2)2\cdot}) = 0$. However, unless the average abundance of all 75th percentile taxa is same between the two ecosystems, i.e. $\tilde{\mu}_{\cdot(1)1} - \tilde{\mu}_{\cdot(2)2} = 0$, the ELib-UQ estimator does not estimate the bias term in the null hypothesis unbiasedly, i.e. $E(\hat{\delta}^{\text{ELib-UQ}}) \neq \delta$.

Thus the ELib-UQ normalization method used in edgeR does not always eliminate the bias due to variability in the sampling fraction. Consequently, the test statistic used in edgeR intrinsically tests a biased hypothesis and hence from Lemma 0.1, it inflates the false positive rate.

We skip the proofs for TMM and ELib-TMM since the arguments are similar.

metagenomeSeq

Suppose the zero-inflated Gaussian (ZIG) mixture model is used in metagenomeSeq. The framework can be summarized as

$$\begin{aligned}& y_{ijk} = \log_2(O_{ijk} + 1) \\ & f_{\text{zig}}(y_{ijk}; O_{\cdot jk}, \mu_{ij}, \sigma_{ij}^2) = \pi_{jk}(O_{\cdot jk}) I_{\{0\}}(y_{ijk}) + (1 - \pi_{jk}(O_{\cdot jk}))\phi(y_{ijk}; \mu_{ij}, \sigma_{ij}^2) \\ & E(y_{ijk}|j=1) = \pi_{jk} \cdot 0 + (1 - \pi_{jk}) \cdot (\beta_{i0} + \eta_i \log_2(\frac{s_{jk}^{\hat{l}} + 1}{N}) + \beta_{i1} I(j=1))\end{aligned} \tag{21}$$

where

(1) $N =$ an approximately choose normalization constant,

(2) $O_{\cdot jk} = \sum_{i=1}^{m} O_{ijk}$ is the library size for sample k in group j,

(3) $s_{jk}^{\hat{l}} = \sum_{i: O_{ijk} \leq q_{jk}^{\hat{l}}} O_{ijk}$,

(4) $q_{jk}^{\hat{l}} = \hat{l}^{th}$ quantile of sample k in group j.

$\hat{l}$ is determined by the smallest l that satisfies

$$\Delta_q^{l+1} - \Delta_q^l \geq 0.1 \Delta_q^l \tag{22}$$

where

$$\begin{aligned}\Delta_q^l &= \text{median}_{jk} |q_{jk}^l - \bar{q}^l| \\ \bar{q}^l &= \text{median}_{jk} q_{jk}^l\end{aligned} \tag{23}$$

The null hypothesis under metagenomeSeq is as follows:

$$\begin{aligned}H_0 &: \beta_{i1} = 0 \\ H_1 &: \beta_{i1} \neq 0\end{aligned}$$

For simplicity of exposition, suppose $\pi_{jk} = 0$. Comparing the ZIG model (21) with the regression framework of ANCOM-BC, we define:

$$\hat{d}_{jk}^{\text{CSS}} = \log(s_{jk}^{\hat{l}} + 1) \tag{24}$$

Hence,

$$\begin{aligned}
\hat{d}_{jk}^{\text{CSS}} &= \log(s_{jk}^{\hat{l}}+1) \\
&\approx \log(s_{jk}^{\hat{l}}) \quad (s_{jk}^{\hat{l}} \text{ is much larger than 1}) \\
&\approx \log(E(s_{jk}^{\hat{l}})) + \frac{1}{E(s_{jk}^{\hat{l}})}(s_{jk}^{\hat{l}} - E(s_{jk}^{\hat{l}})) \quad \text{(Taylor's expansion)} \\
&= \log(\sum_{i:O_{ijk}\leq q_{jk}^{\hat{l}}} c_{jk}\theta_{ij}) + \frac{s_{jk}^{\hat{l}}}{E(s_{jk}^{\hat{l}})} - 1 \\
&= d_{jk} + \log(\sum_{i:O_{ijk}\leq q_{jk}^{\hat{l}}} \theta_{ij}) + \frac{s_{jk}^{\hat{l}}}{E(s_{jk}^{\hat{l}})} - 1 \\
&:= d_{jk} + x_{a_{jk}j} + z_{jk} - 1
\end{aligned} \tag{25}$$

As before, for the kth sample in the jth group, a_{jk} represents the index such that $\log(\sum_{i:O_{ijk}\leq q_{jk}^{\hat{l}}} \theta_{ij}) = x_{a_{jk}j}$, and $z_{jk} := \frac{s_{jk}^{\hat{l}}}{E(s_{jk}^{\hat{l}})}$.

Averaging over all sample $k = 1, 2, \ldots n$, we get

$$\bar{\hat{d}}_{j\cdot}^{\text{CSS}} = d_{j\cdot} + \tilde{x}_{\cdot(j)j} + \bar{z}_{j\cdot} - 1 \tag{26}$$

Since each subject k in group j, may potentially have a different total mean absolute abundance up to the $\hat{l}^{th}$ percentile, in the above expression $\tilde{x}$ represents the mean of variable x taken over the suitable subset of taxa. Secondly, the notation $\cdot(j)$ represents the mean taken within group j. $\bar{z}_{j\cdot}$ is the average of z_{jk}.

Thus, the CSS estimator of the bias term in the null hypothesis is:

$$\begin{aligned}
\hat{\delta}^{\text{CSS}} &:= \bar{\hat{d}}_{1\cdot}^{\text{CSS}} - \bar{\hat{d}}_{2\cdot}^{\text{CSS}} \\
&= \bar{d}_{1\cdot} - \bar{d}_{2\cdot} + (\tilde{x}_{\cdot(1)1} - \tilde{x}_{\cdot(2)2}) + (\bar{z}_{1\cdot} - \bar{z}_{2\cdot}) \\
&= \delta + (\tilde{x}_{\cdot(1)1} - \tilde{x}_{\cdot(2)2}) + (\bar{z}_{1\cdot} - \bar{z}_{2\cdot})
\end{aligned} \tag{27}$$

Note that unless $\tilde{x}_{\cdot(1)1} - \tilde{x}_{\cdot(2)2} = 0$, which means the sum up to $\hat{l}^{th}$ percentile of the mean absolute abundance is the same between two groups, the CSS estimator does not estimate the bias term in the null hypothesis unbiasedly, i.e. $E(\hat{\delta}^{\text{CSS}}) \neq \delta$.

Therefore, although metagenomeSeq directly tests for differential absolute abundance, there is a systematic bias in estimating sampling fractions. Again, according to Lemma 0.1, it suffers from inflated FDR as well.

Residual analysis of normalization methods for differential sampling fractions

Although not explicitly stated, each normalization method available in the literature, such as the Cumulative-Sum Scaling (CSS) implemented in metagenomeSeq [4], Median (MED) in DESeq2 [2], Upper Quartile (UQ), Trimmed Mean of M-values (TMM), Total-Sum Scaling (TSS), as well as the modifications of UQ and TMM, denoted by ELib-UQ and ELib-TMM used in edgeR [3], that account for "Effective Library size" [5], attempt to normalize the data for variability in sampling fractions across samples. In this section we describe a simple method to evaluate the performance of some of these available normalization methods, along with our proposed method in ANCOM-BC.

Suppose we have two experimental groups with balanced sample size, for each normalization method s, sample $k = 1, 2, \ldots, n$, in the jth group, $j = 1, 2$, let the (raw) residual be denoted by

$$r_{jk}^{s} = \hat{d}_{jk}^{s} - d_{jk}. \tag{28}$$

Then $\bar{r}_{j\cdot}^{s} = \bar{\hat{d}}_{j\cdot}^{s} - \bar{d}_{j\cdot}$, therefore, $\bar{r}_{1\cdot}^{s} - \bar{r}_{2\cdot}^{s} = (\bar{\hat{d}}_{1\cdot}^{s} - \bar{\hat{d}}_{2\cdot}^{s}) - (\bar{d}_{1\cdot} - \bar{d}_{2\cdot})$. Since residuals generated by each normalization method will have their own center, to align the box plot of residuals at the same level, we center the raw residuals by

$$r_{jk}^{s*} = r_{jk}^{s} - \bar{r}_{\cdot\cdot}^{s} = \hat{d}_{jk}^{s} - d_{jk} - \bar{\hat{d}}_{\cdot\cdot}^{s} + \bar{d}_{\cdot\cdot}. \tag{29}$$

and make box plots using these (centered) residuals. Thus, if the normalization method is effective then there should be no systematic pattern among the residuals by the experimental groups. Otherwise, the normalization method is not successfully eliminating the bias due to variability in sampling fractions.

Based on our simulated data (Fig. 3, Supplementary Fig. 1, 2), as expected ANCOM-BC seems to successfully eliminate the bias induced by the differences in the sampling fractions between two experimental groups. For ANCOM-BC, the samples from the two groups (circles and triangles) are nicely intermixed with small variability of residuals. Consistent with our observations in the previous section, this is not always the case with other methods. For other methods, the group labels are not randomly distributed around zero but they are clustered by the group label (Fig. 3, Supplementary Fig. 1). This suggests that the existing normalization methods do not eliminate the systematic bias introduced by the differences in the sampling fractions.

Estimators of sampling fractions by different normalization methods are summarized in Supplementary Table 7.

Compositional structure of RNA-Seq data

Similar to microbiome data, RNA-Seq data are intrinsically compositional [6, 7]. This is due to the limitation of high-throughput sequencing (HTS) experiments. The sequencing instruments can deliver reads only up to their capacity, which is a fixed number of slots that are able to be filled [8]. DESeq2 and edgeR try to get around with the compositional structure by scaling the raw data using some normalization factors. For instance, as stated in the user manual of edgeR [5], the authors realize that the highly expressed genes can occupy a substantial proportion of the library size, causing the remaining genes to be under-expressed in that sample. Therefore, to address the "RNA composition" effect, they first scale the raw data and then replace the original library size with the effect library size.

We thus conclude that both RNA-Seq data and microbiome data are compositional. Based on our extensive simulation studies and real data analyses, we believe the most proper way to deal with the compositional effect is by estimating and correcting for the difference of sampling fractions directly.

Supplementary Notes

Simulation settings

Denote $\mathcal{M}_0$ = the set of non-differentially abundant taxa, $\mathcal{M}_1$ = the set of differentially abundant taxa, $\mathcal{M}_1 = \mathcal{M}_0^c$, $\mathcal{T}_0$ =the set of non-differentially abundant taxa identified by DA analyses, $\mathcal{T}_1$ =the set of differentially abundant taxa identified by DA analyses, $\mathcal{T}_1 = \mathcal{T}_0^c$. See Supplementary Fig. 11-14 for simulation flowcharts.

Fig. 3

(a) Nominal level = 0.05

(b) Number of simulations = 1

(c) Sample size: $n_1 = 30, n_2 = 30$

(d) Number of taxa: $m = 500$

(e) Proportion of differentially abundant taxa = 25%

(f) Proportion of structure zeros = 0% out of non-differentially abundant taxa

(g) Proportion of outlier zeros = 0% out of samples

(h) Mean absolute abundance in the ecosystem: $\theta_{ij} \sim \text{GAM}(a, 1)$, where $a = 50$ represents low abundant taxa, $a = 200$ represents medium abundant taxa, and $a = 10,000$ represents high abundant taxa.

  (i) The proportions of low, median, and high abundant taxa are set to be $60\%, 30\%, 10\%$

  (ii) The effect size α_i for differentially abundant taxa is set to follow $\text{U}(0.1, 1) \cup \text{U}(1, 10)$ and apply to θ_{i1}. This leads to **unbalanced** microbial loads

(i) (Unobserved) absolute abundance in the ecosystem: $A_{ijk}|\theta_{ij} \sim \text{POI}(\theta_{ij})$

(j) (Observed) absolute abundance in a sample:

  (i) **Balanced** library size across groups: $O_{\cdot jk} = p_{jk} \max(A_{\cdot jk})$, where $p_{jk} \sim \frac{1}{\text{U}(5,10)}$

  (ii) $O_{ijk} \sim \text{BIN}(O_{\cdot jk}, \gamma_{ijk} = \frac{A_{ijk}}{A_{\cdot jk}})$

Fig. 4

Simulation settings are the same as Fig. 3 except that:

(b) Number of simulations = 100

(c) Sample size

  (i) $n_1 = 20, n_2 = 30$

  (ii) $n_1 = n_2 = 50$

(d) Number of taxa: $m = 1000$

(e) Proportion of differentially abundant taxa = $5\%, 15\%, 25\%$

(f) Proportion of structure zeros = 20% out of non-differentially abundant taxa

(g) Proportion of outlier zeros = 5% out of samples

(j) (Observed) absolute abundance in a sample:

  (i) **Balanced** library size across groups: $O_{\cdot jk} = p_{jk} \max(A_{\cdot jk})$, where $p_{jk} \sim \frac{1}{\text{U}(10,50) \cup \text{U}(100,500)}$

  (ii) $O_{ijk} \sim \text{BIN}(O_{\cdot jk}, \gamma_{ijk} = \frac{A_{ijk}}{A_{\cdot jk}})$

Supplementary Fig. 1

Simulation settings are the same as Fig. 3 except that:

(j) (Observed) absolute abundance in a sample:

  (i) **Unbalanced** library size across groups: $O_{\cdot jk} = p_{jk} A_{\cdot jk}$, where $p_{jk} \sim \frac{1}{\text{U}(5,10)}$

  (ii) $O_{ijk} \sim \text{BIN}(O_{\cdot jk}, \gamma_{ijk} = \frac{A_{ijk}}{A_{\cdot jk}})$

Supplementary Fig. 2

Simulation settings are the same as Fig. 3 except that:

(h) Mean absolute abundance in the ecosystem: $\theta_{ij} \sim \text{GAM}(a, 1)$, where $a = 50$ represents low abundant taxa, $a = 200$ represents medium abundant taxa, and $a = 10,000$ represents high abundant taxa.

  (i) The proportions of low, median, and high abundant taxa are set to be $60\%, 30\%, 10\%$

  (ii) The effect size α_i for differentially abundant taxa is set to be $\text{U}(1, 10)$ and apply to **both** θ_{i1} **and** θ_{i2}. This leads to **balanced** microbial loads

Supplementary Fig. 3

Simulation settings are the same as Fig. 4 except that:

(j) (Observed) absolute abundance in a sample:

  (i) **Unbalanced** library size across groups: $O_{\cdot jk} = p_{jk} A_{\cdot jk}$, where $p_{jk} \sim \frac{1}{\text{U}(10,50) \cup \text{U}(100,500)}$

  (ii) $O_{ijk} \sim \text{BIN}(O_{\cdot jk}, \gamma_{ijk} = \frac{A_{ijk}}{A_{\cdot jk}})$

Supplementary Fig. 4

Simulation settings are the same as Fig. 4 except that:

(h) Mean absolute abundance in the ecosystem: $\theta_{ij} \sim \text{GAM}(a, 1)$, where $a = 50$ represents low abundant taxa, $a = 200$ represents medium abundant taxa, and $a = 10,000$ represents high abundant taxa.

  (i) The proportions of low, median, and high abundant taxa are set to be $60\%, 30\%, 10\%$

  (ii) The effect size α_i for differentially abundant taxa is set to be $\text{U}(1, 10)$ and apply to **both** θ_{i1} **and** θ_{i2}. This leads to **balanced** microbial loads

Supplementary Fig. 5

Simulation settings are the same as Fig. 4 except that:

(h) Mean absolute abundance in the ecosystem: θ_{ij} is from the absolute abundance of soil samples of global pattern data [9]

(i) (Unobserved) absolute abundance in the ecosystem: $A_{ijk}|\theta_{ij} = \theta_{ij}$

Supplementary Fig. 6

Simulation settings are the same as Fig. 4 except that:

(c) Sample size: $n_1 = 5, n_2 = 5$ or $n_1 = 10, n_2 = 10$

Supplementary Fig. 7

Simulation settings are the same as Fig. 4 except that:

(e) Proportion of differentially abundant taxa = 50% and 75%

Supplementary Fig. 8

Simulation settings are the same as Fig. 3 except that:

(f) Proportion of structure zeros = 20% out of non-differentially abundant taxa

(g) Proportion of outlier zeros = 5% out of samples

(j) (Observed) absolute abundance in a sample:
   (i) **Balanced** library size across groups: $O_{\cdot jk} = p_{jk} \max(A_{\cdot jk})$, where $p_{jk} \sim \frac{1}{\mathrm{U}(10,50) \cup \mathrm{U}(100,500)}$

Supplementary Fig. 9

Simulation settings are the same as Fig. 3 except that:

(e) Proportion of differentially abundant taxa = 5%, 15%, 25%

(f) Proportion of structure zeros = 20% out of non-differentially abundant taxa

(g) Proportion of outlier zeros = 5% out of samples

(j) (Observed) absolute abundance in a sample:
   (i) **Balanced** library size across groups: $O_{\cdot jk} = p_{jk} \max(A_{\cdot jk})$, where $p_{jk} \sim \frac{1}{\mathrm{U}(10,50) \cup \mathrm{U}(100,500)}$

Supplementary Fig. 10

Simulation settings are the same as Fig. 4 except that BH procedure was implemented for every differential abundance (DA) method for adjustments of multiple comparisons.

Supplementary Table 4

Simulation settings are the same as Fig. 4 except that:

(d) Number of taxa: $m = 10$ or $m = 50$

(h) Mean absolute abundance in the ecosystem: $\theta_{ij} \sim \text{GAM}(a, 1)$, where

  (1) When $m = 10$: $a = 5,000$ represents low abundant taxa, $a = 20,000$ represents medium abundant taxa, and $a = 1,000,000$ represents high abundant taxa.

  (2) When $m = 50$: $a = 500$ represents low abundant taxa, $a = 2,000$ represents medium abundant taxa, and $a = 100,000$ represents high abundant taxa.

Supplementary Table 5

(a) Nominal level = 0.05

(b) Number of simulations = 100

(c) Sample size

  (i) $n_1 = n_2 = 20, n_3 = n_4 = 30$

  (ii) $n_1 = n_2 = n_3 = n_4 = 50$

(d) Number of taxa: $m = 1000$

(e) Proportion of differentially abundant taxa = $5\%, 15\%, 25\%$

(f) Proportion of structure zeros = 20% out of non-differentially abundant taxa

(g) Proportion of outlier zeros = 5% out of samples

(h) The reference group: group 1

(i) Mean absolute abundance in the ecosystem: $\theta_{ij} \sim \text{GAM}(a, 1)$, where $a = 50$ represents low abundant taxa, $a = 200$ represents medium abundant taxa, and $a = 10,000$ represents high abundant taxa.

  (i) The proportions of low, median, and high abundant taxa are set to be $60\%, 30\%, 10\%$

  (ii) For group 2, 3, and 4, randomly choose which is/are differentially abundant with group 1

  (iii) The effect size α_i:

  - group 1 = 1
  - group $j, j \in \{2, 3, 4\} \sim \text{U}(0.1, 1) \cup \text{U}(1, 10)$

(j) (Unobserved) absolute abundance in the ecosystem: $A_{ijk} | \theta_{ij} \sim \text{POI}(\theta_{ij})$

(k) (Observed) absolute abundance in a sample:

  (i) Library size: $O_{\cdot jk} = p_{jk} A_{\cdot jk}$, where $p_{jk} \sim \frac{1}{\text{U}(10,50) \cup \text{U}(100,500)}$

  (ii) $O_{ijk} \sim \text{BIN}(O_{\cdot jk}, \gamma_{ijk} = \frac{A_{ijk}}{A_{\cdot jk}})$

Supplementary Table 6

Simulation settings are the same as Fig. 4 except that:

(c) Sample size: $n_1 = n_2 = 50$

Supplementary Figures

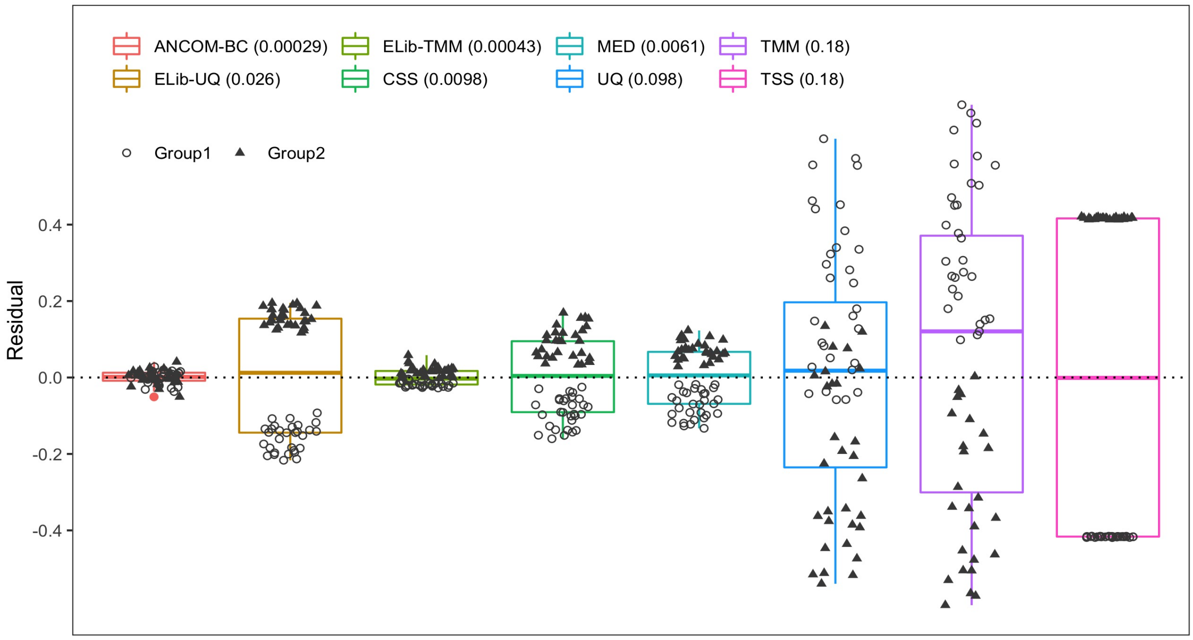

Supplementary Figure 1: Box plot of residuals between true sampling fraction and its estimate for each sample.

In the box plot, the lower and upper hinges correspond to the first and third quartiles (the 25th and 75th percentiles). The median is represented by a solid line within the box. The upper whisker extends from the hinge to the largest value (maxima) no further than 1.5 times Interquartile Range (IQR, distance between the first and third quartiles) from the hinge, the lower whisker extends from the hinge to the smallest value (minima) at most 1.5 times IQR of the hinge. Data beyond the end of the whiskers are called "outlying" points. N = 30 samples examined over 2 experimental groups (denoted by circles and triangles) and the data points are overlaid in each box. Text on the upper left corner indicates the color for each method and variances are provided within parenthesis for each method. The variability in sampling fractions is set to be moderate. ANCOM-BC has the smallest variance, while TMM and TSS have the largest. Except ANCOM-BC, UQ, and TMM, all remaining methods show certain degree of group separation of residuals. Compared to Fig. 3, the variance and separation of residuals shown by ELib-UQ, ELib-TMM, CSS, and MED are slightly reduced since the variability of sampling fractions is smaller in this case.

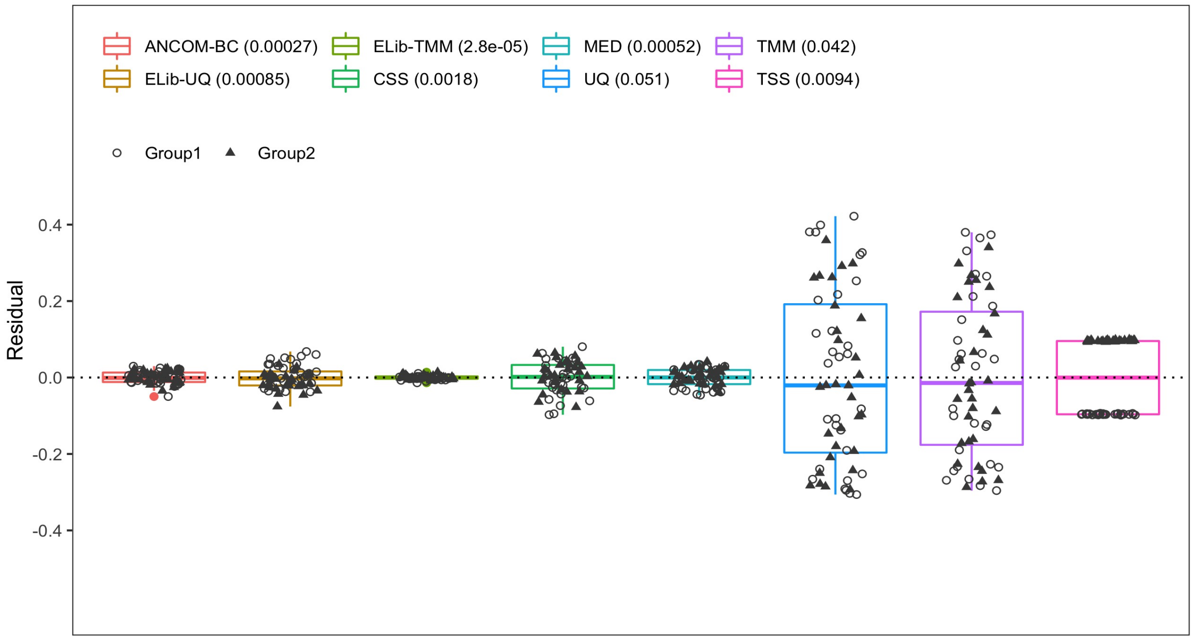

Supplementary Figure 2: Box plot of residuals between true sampling fraction and its estimate for each sample.

In the box plot, the lower and upper hinges correspond to the first and third quartiles (the 25th and 75th percentiles). The median is represented by a solid line within the box. The upper whisker extends from the hinge to the largest value (maxima) no further than 1.5 times Interquartile Range (IQR, distance between the first and third quartiles) from the hinge, the lower whisker extends from the hinge to the smallest value (minima) at most 1.5 times IQR of the hinge. Data beyond the end of the whiskers are called "outlying" points. N = 30 samples examined over 2 experimental groups (denoted by circles and triangles) and the data points are overlaid in each box. Text on the upper left corner indicates the color for each method and variances are provided within parenthesis for each method. The variability in sampling fractions is set to be small. ELib-TMM has the smallest variance, while UQ has the largest. ANCOM-BC competes well with ELib-TMM regarding the variance. Except TSS, the separation of residuals shown by remaining methods is practically non-existent since both library sizes and microbial loads are balanced, and the variability of sampling fractions is small in this case.

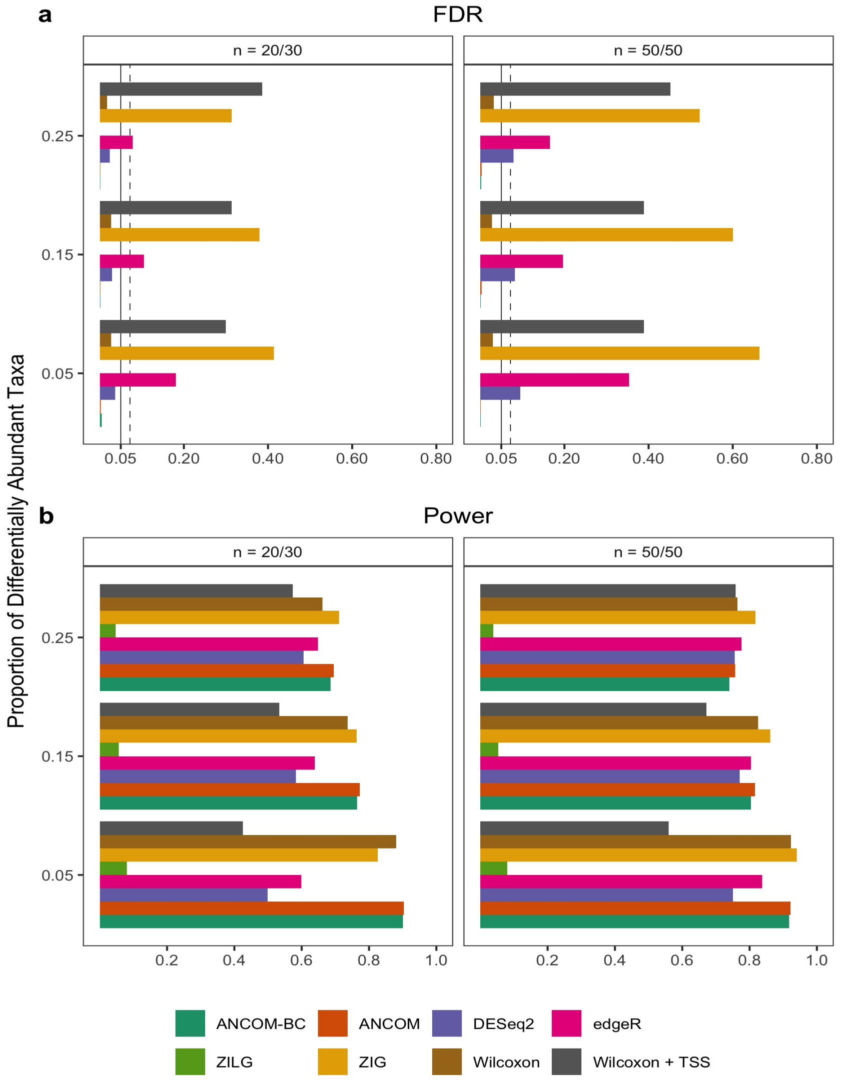

Supplementary Figure 3: FDR and power comparisons using synthetic data from Poisson-Gamma distributions.

The False Discovery Rate (FDR) and power of various differential abundance (DA) analyses (two-sided) are shown in panel **a** and panel **b**, respectively. The variability in sampling fractions is set to be moderate. The Y-axis denotes patterns of proportion of differentially abundant taxa. The solid vertical line is the 5% nominal level of FDR, and the dashed vertical line denotes 5% nominal level plus one standard error (SE). By default, ANCOM-BC implements Bonferroni correction and other DA methods implement BH procedure to adjust for multiple comparisons. Color and the name of the corresponding DA method are shown at the bottom within the graph. Two simulation scenarios are considered: small and unbalanced data ($n_1 = 20$, $n_2 = 30$), as well as large and balanced data ($n_1 = n_2 = 50$); number of simulations = 100. Results show that ANCOM, ANCOM-BC and the simple Wilcoxon test control the FDR under the nominal level (5%) while maintaining power comparable to other methods in this simulation setting. Gaussian model version of metagenomeSeq has highly inflated FDR, while the Log-Gaussian version has substantial loss of power, sometimes well below 5%. Other than ANCOM-BC and ANCOM, as the sample size within each group increases, so does the FDR for all other existing

methods.

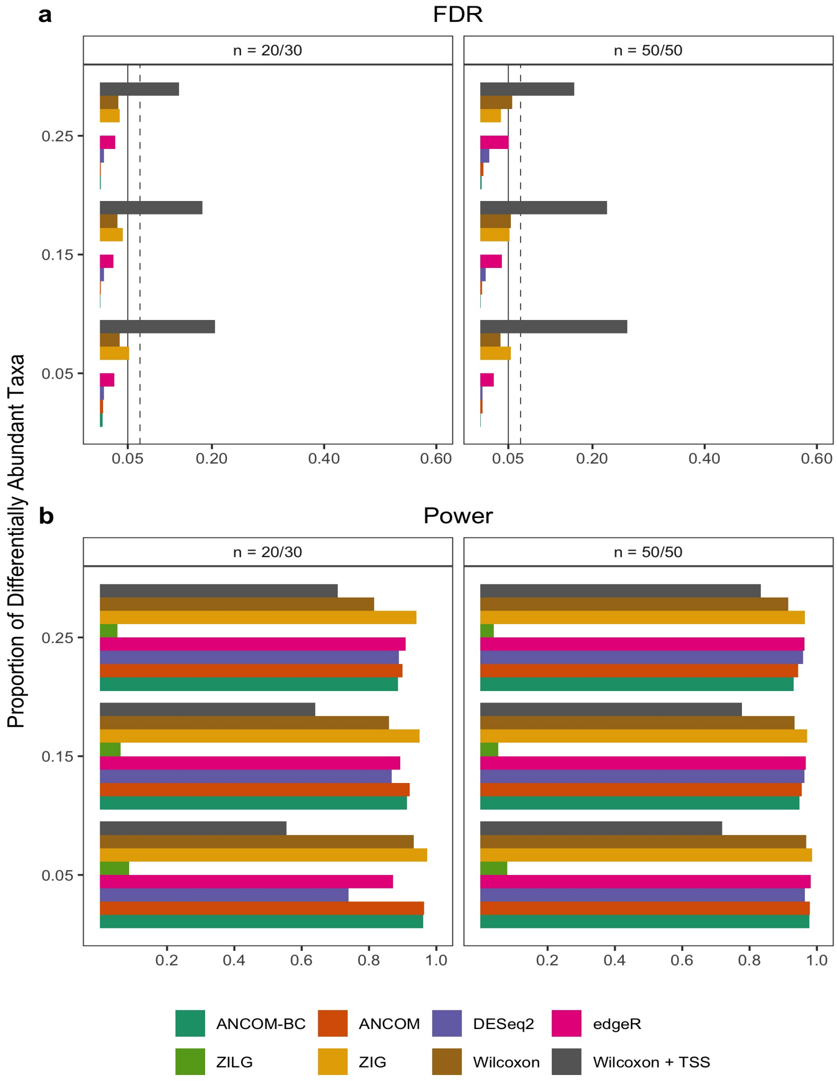

Supplementary Figure 4: FDR and power comparisons using synthetic data from Poisson-Gamma distributions.

The False Discovery Rate (FDR) and power of various differential abundance (DA) analyses (two-sided) are shown in panel **a** and panel **b**, respectively. The variability in sampling fractions is set to be small. The Y-axis denotes patterns of proportion of differentially abundant taxa. The solid vertical line is the 5% nominal level of FDR, and the dashed vertical line denotes 5% nominal level plus one standard error (SE). By default, ANCOM-BC implements Bonferroni correction and other DA methods implement BH procedure to adjust for multiple comparisons. Color and the name of the corresponding DA method are shown at the bottom within the graph. Two simulation scenarios are considered: small and unbalanced data ($n_1 = 20$, $n_2 = 30$), as well as large and balanced data ($n_1 = n_2 = 50$); number of simulations = 100. Results show that all DA analyses except Wilcoxon test with TSS control the FDR under the nominal level (5%) while maintaining comparable power to each other in this simulation setting. The Log-Gaussian version of metagenomeSeq has substantial loss of power, sometimes well below 5%. Other than ANCOM-BC and ANCOM, as the sample size within each group increases, so does the FDR for all other existing methods.

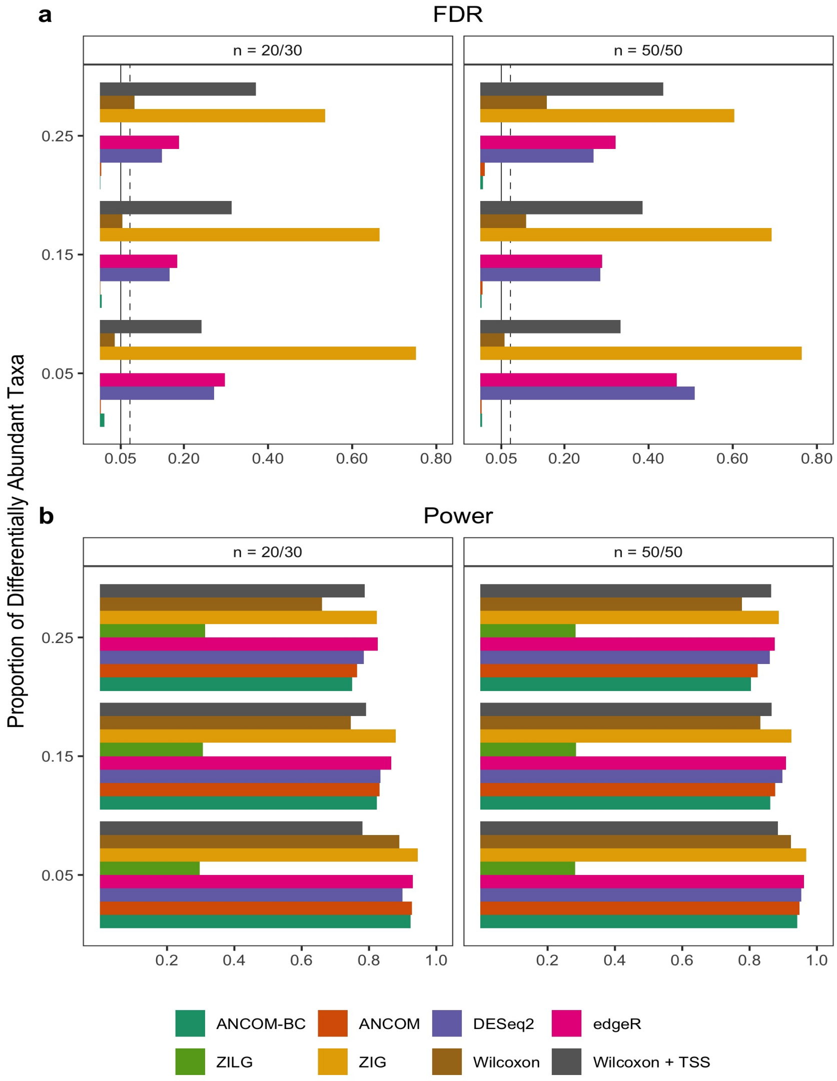

Supplementary Figure 5: FDR and power comparisons using synthetic data from soil samples of global pattern data [9].

The False Discovery Rate (FDR) and power of various differential abundance (DA) analyses (two-sided) are shown in panel **a** and panel **b**, respectively. The variability in sampling fractions is set to be large. The Y-axis denotes patterns of proportion of differentially abundant taxa. The solid vertical line is the 5% nominal level of FDR, and the dashed vertical line denotes 5% nominal level plus one standard error (SE). By default, ANCOM-BC implements Bonferroni correction and other DA methods implement BH procedure to adjust for multiple comparisons. Color and the name of the corresponding DA method are shown at the bottom within the graph. Two simulation scenarios are considered: small and unbalanced data ($n_1 = 20$, $n_2 = 30$), as well as large and balanced data ($n_1 = n_2 = 50$); number of simulations = 100. The results are similar to Fig. 4a, b shown in the main text except the observation of increasing FDR for DESeq2 and edgeR since the data no longer follow the Poisson-Gamma distribution.

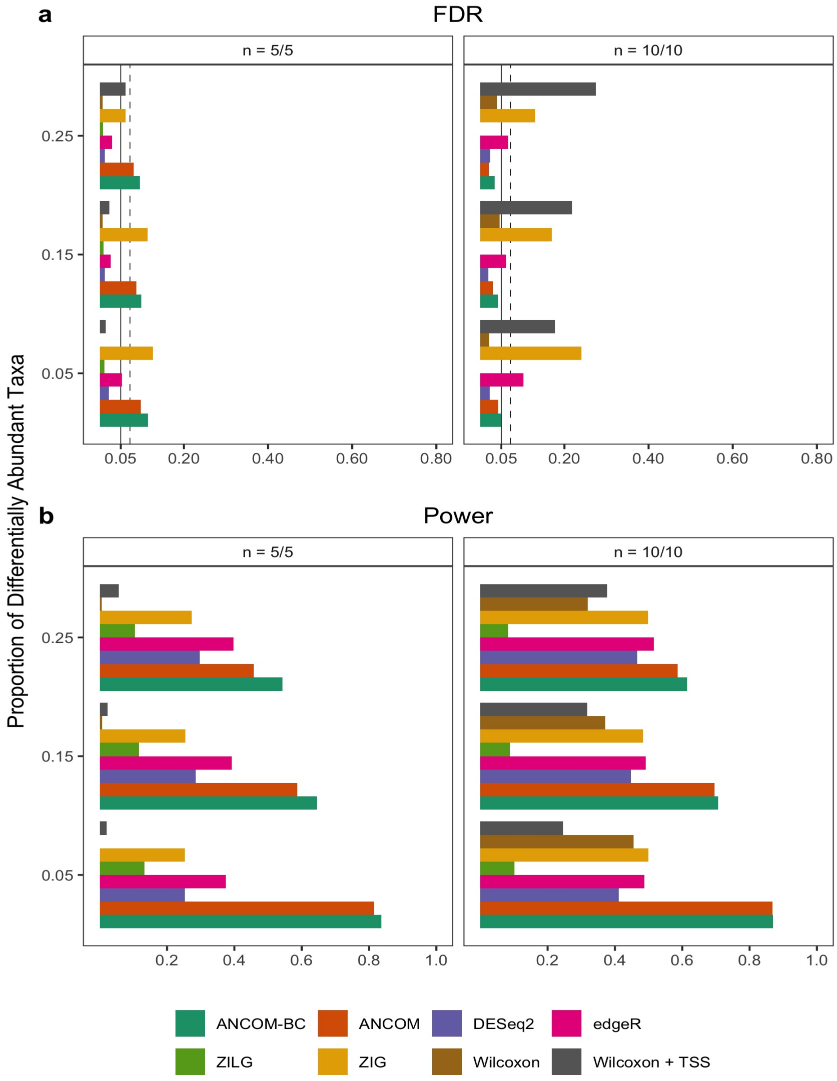

Supplementary Figure 6: FDR and power comparisons when sample size is small

The False Discovery Rate (FDR) and power of various differential abundance (DA) analyses (two-sided) are shown in panel **a** and panel **b**, respectively. Data are generated from Poisson-Gamma distributions. The variability in sampling fractions is set to be large. The Y-axis denotes patterns of proportion of differentially abundant taxa. The solid vertical line is the 5% nominal level of FDR, and the dashed vertical line denotes 5% nominal level plus one standard error (SE). By default, ANCOM-BC implements Bonferroni correction and other DA methods implement BH procedure to adjust for multiple comparisons. Color and the name of the corresponding DA method are shown at the bottom within the graph. Two simulation scenarios are considered: $n_1 = n_2 = 5$ and $n_1 = n_2 = 10$; number of simulations = 100. ANCOM-BC loses control of FDR when the sample size is extremely small (5 per group) while it manages to control FDR as the sample size increases to 10 per group. ANCOM-BC has the largest power among all DA analyses.

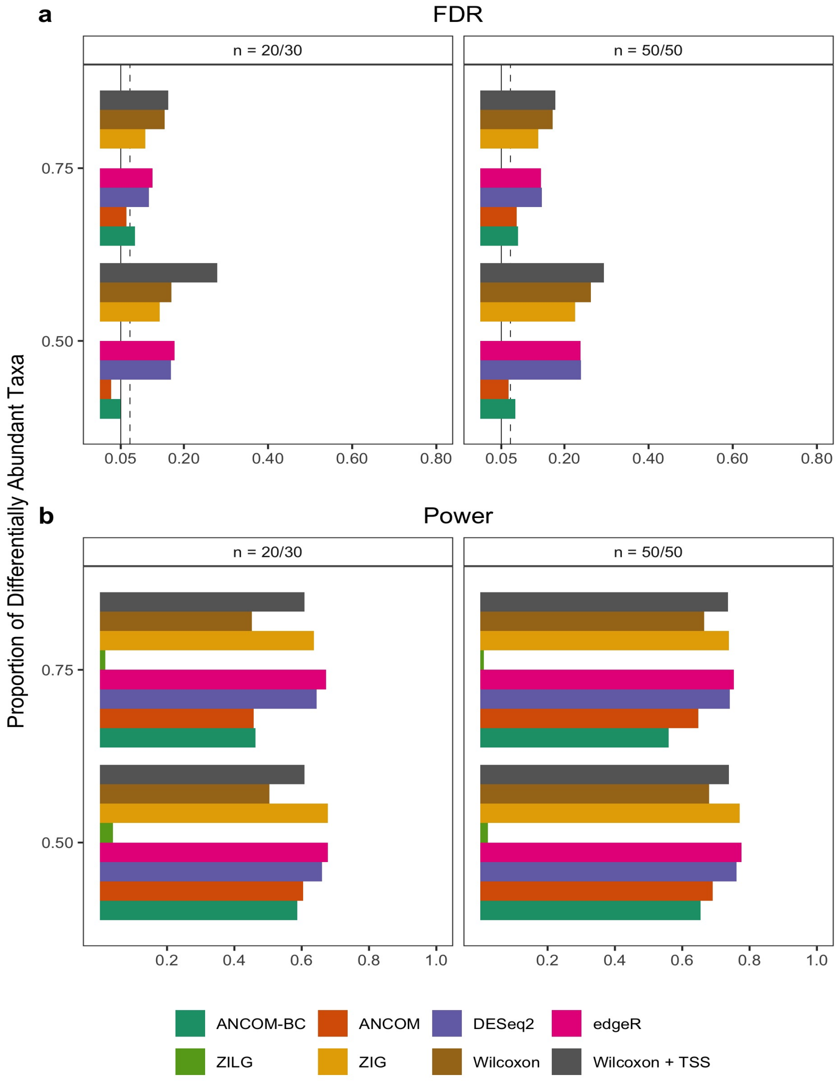

Supplementary Figure 7: FDR and power comparisons when the proportion of differentially abundant taxa is large.

The False Discovery Rate (FDR) and power of various differential abundance (DA) analyses (two-sided) are shown in panel **a** and panel **b**, respectively. Data are generated from Poisson-Gamma distributions. The variability in sampling fractions is set to be large. The Y-axis denotes patterns of proportion of differentially abundant taxa. The solid vertical line is the 5% nominal level of FDR, and the dashed vertical line denotes 5% nominal level plus one standard error (SE). By default, ANCOM-BC implements Bonferroni correction and other DA methods implement BH procedure to adjust for multiple comparisons. Color and the name of the corresponding DA method are shown at the bottom within the graph. Two simulation scenarios are considered: small and unbalanced data ($n_1 = 20$, $n_2 = 30$), as well as large and balanced data ($n_1 = n_2 = 50$); number of simulations = 100. ANCOM-BC requires a certain number of non-differentially abundant taxa for precise estimates of sampling fractions. When the proportion of differentially abundant taxa is large (e.g. 75%), ANCOM-BC slightly exceeds the nominal level with regard to FDR.

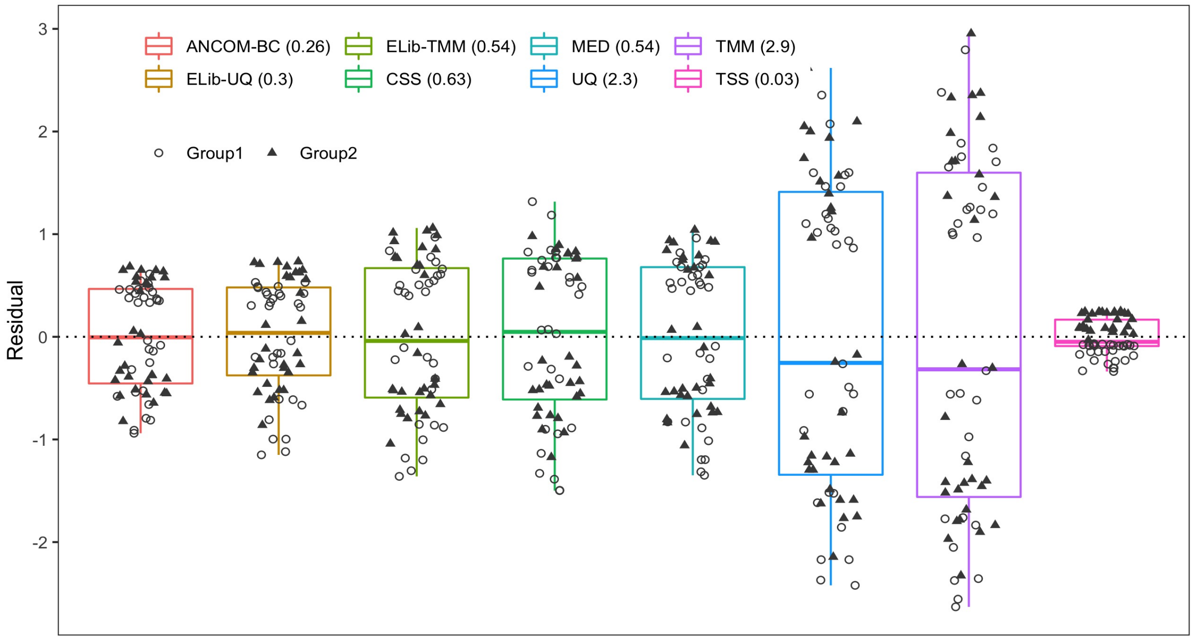

Supplementary Figure 8: Box plot of residuals between true sampling fraction and its estimate for each sample.

In the box plot, the lower and upper hinges correspond to the first and third quartiles (the 25th and 75th percentiles). The median is represented by a solid line within the box. The upper whisker extends from the hinge to the largest value (maxima) no further than 1.5 times Interquartile Range (IQR, distance between the first and third quartiles) from the hinge, the lower whisker extends from the hinge to the smallest value (minima) at most 1.5 times IQR of the hinge. Data beyond the end of the whiskers are called "outlying" points. N = 30 samples examined over 2 experimental groups (denoted by circles and triangles) and the data points are overlaid in each box. Text on the upper left corner indicates the color for each method and variances are provided within parenthesis for each method. The variability in sampling fractions is set to be large. With smaller sampling fractions as compared to the settings of Fig. 3 in the main text, this figure shows that UQ and TMM have the largest variance, ELib-UQ, ELib-TMM, MED and CSS have larger variance than ANCOM-BC, while TSS has the least. However, only TSS shows a clear separation of residuals by its group label, which indicates that TSS has a systematic bias of estimating sampling fractions. Samples from the two groups are inter-mixed well for all the remaining methods in this simulated data.

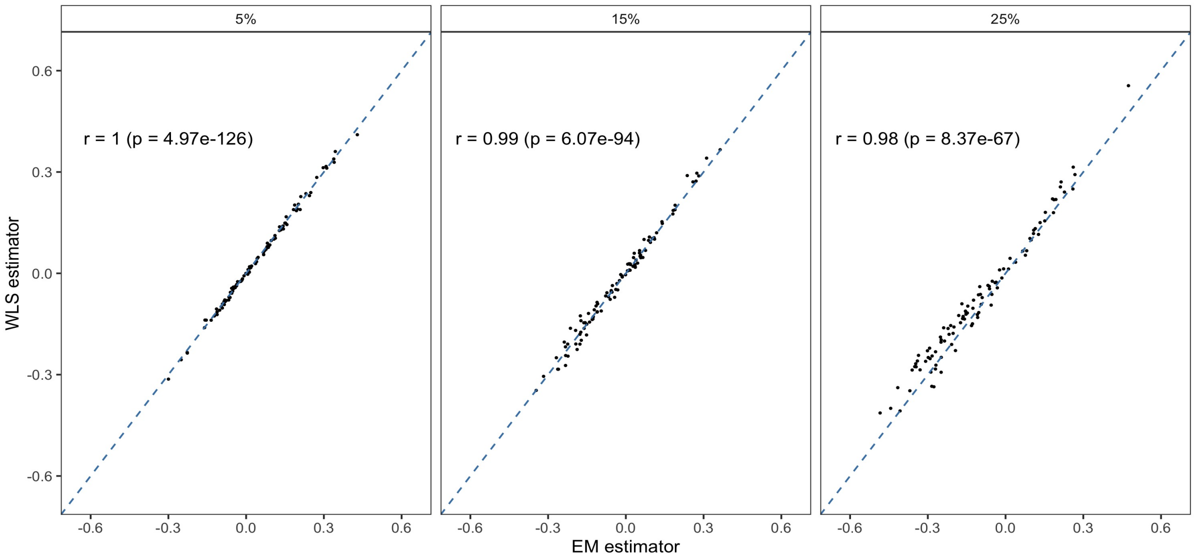

Supplementary Figure 9: EM and weighted least square (WLS) estimators of the bias term are highly correlated.

We computed the Pearson correlation coefficient between $\hat{\delta}_{\text{EM}}$ and $\hat{\delta}_{\text{WLS}}$ along with p-value. The range of differentially abundant taxa was set from 5% to 25% (shown as the panel title). It is clearly that $\hat{\delta}_{\text{EM}}$ is highly correlated with $\hat{\delta}_{\text{WLS}}$ in all simulation scenarios: $r = 1$ ($p = 4.97 \times 10^{-126}$) when 5% of taxa are differentially abundant; $r = 0.99$ ($p = 6.07 \times 10^{-94}$) when 15% of taxa are differentially abundant; $r = 0.98$ ($p = 8.37 \times 10^{-64}$) when 25% of taxa are differentially abundant. Hence, it is reasonable to approximate $\hat{\delta}_{\text{EM}}$ and $\text{Var}(\hat{\delta}_{\text{EM}})$ by $\hat{\delta}_{\text{WLS}}$ and $\text{Var}(\hat{\delta}_{\text{WLS}})$, respectively.

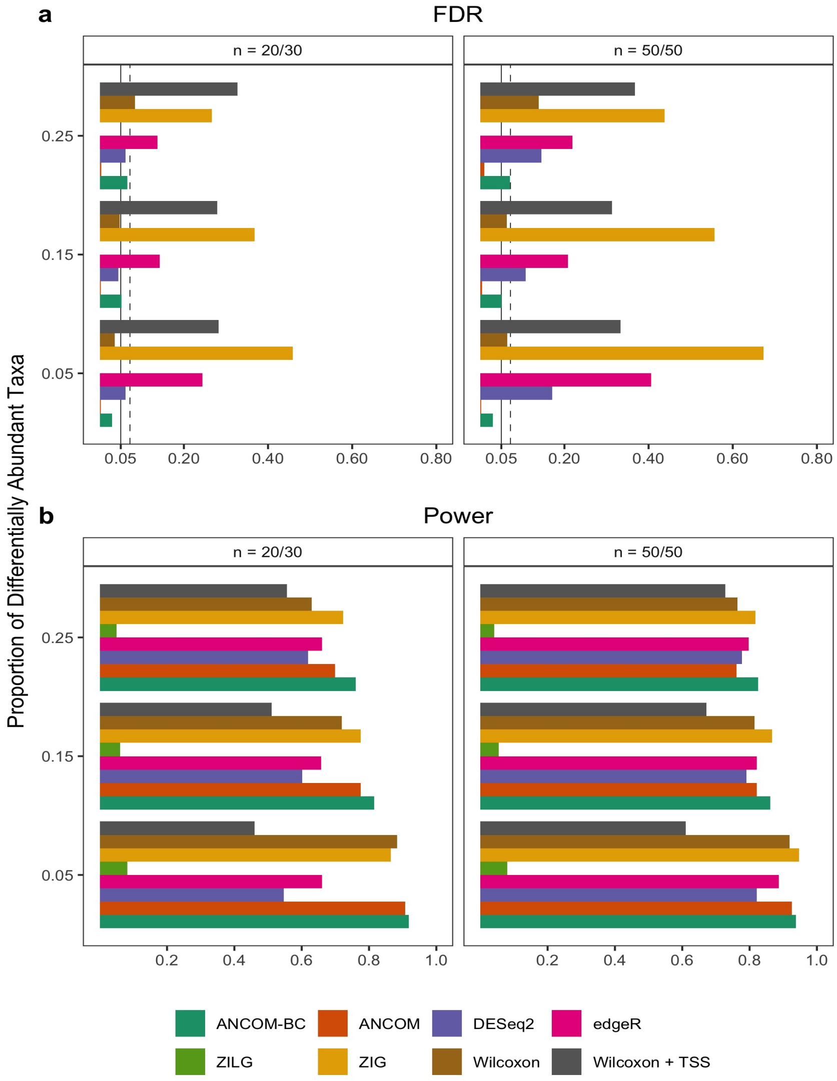

Supplementary Figure 10: FDR and power comparisons using synthetic data from Poisson-Gamma distributions.

The False Discovery Rate (FDR) and power of various differential abundance (DA) analyses (two-sided) are shown in panel **a** and panel **b**, respectively. BH procedure were made for multiple comparisons for all DA methods. The variability in sampling fractions is set to be large. The Y-axis denotes patterns of proportion of differentially abundant taxa. The solid vertical line is the 5% nominal level of FDR, and the dashed vertical line denotes 5% nominal level plus one standard error (SE). Color and the name of the corresponding DA method are shown at the bottom within the graph. Two simulation scenarios are considered: small and unbalanced data ($n_1 = 20$, $n_2 = 30$), as well as large and balanced data ($n_1 = n_2 = 50$); number of simulations $= 100$. Results are similar to those shown in Fig. 4, only ANCOM and ANCOM-BC control the FDR under the nominal level (5%), but ANCOM-BC has the largest power as compared to other methods in this case.

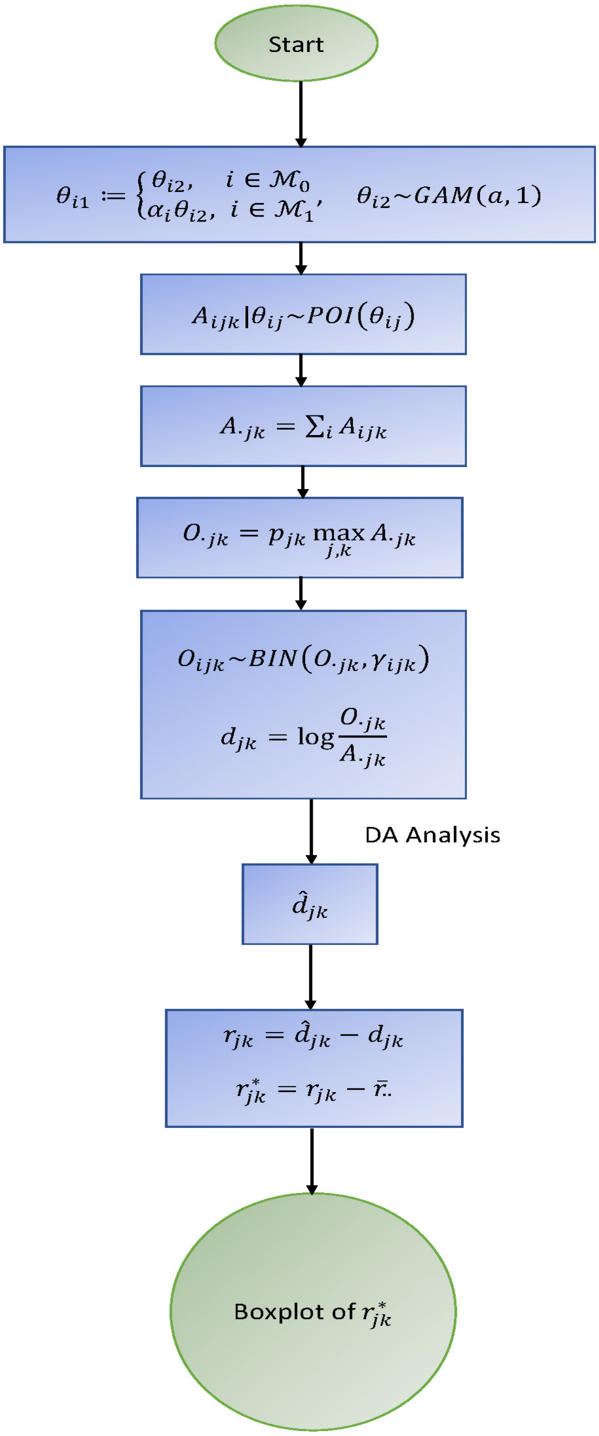

Supplementary Figure 11: Flowchart of simulation for comparing normalization efficacy.

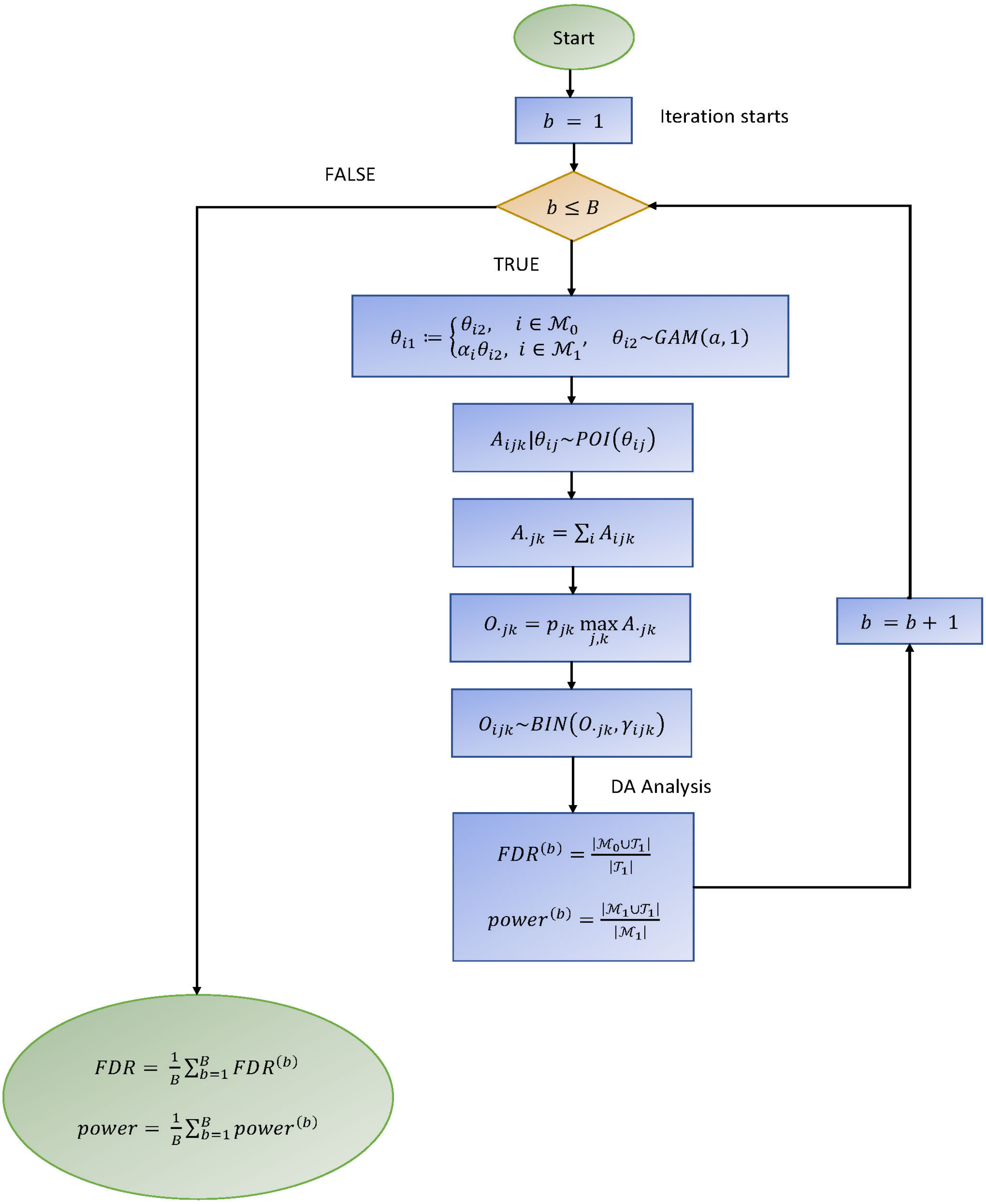

Supplementary Figure 12: Flowchart of simulation for FDR and power evaluation using Poisson-Gamma models: Two-group comparison.

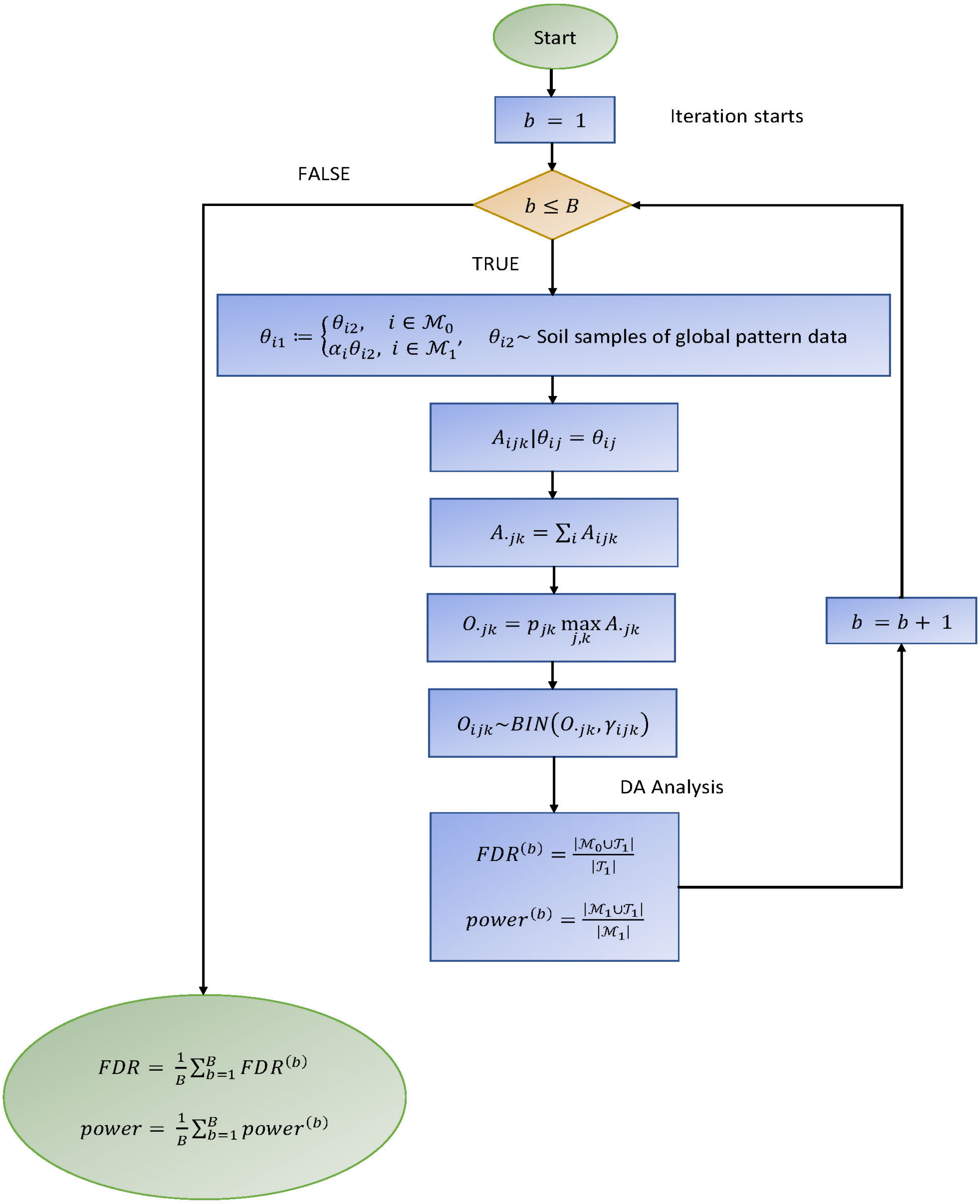

Supplementary Figure 13: Flowchart of simulation for FDR and power evaluation using global pattern data [9]

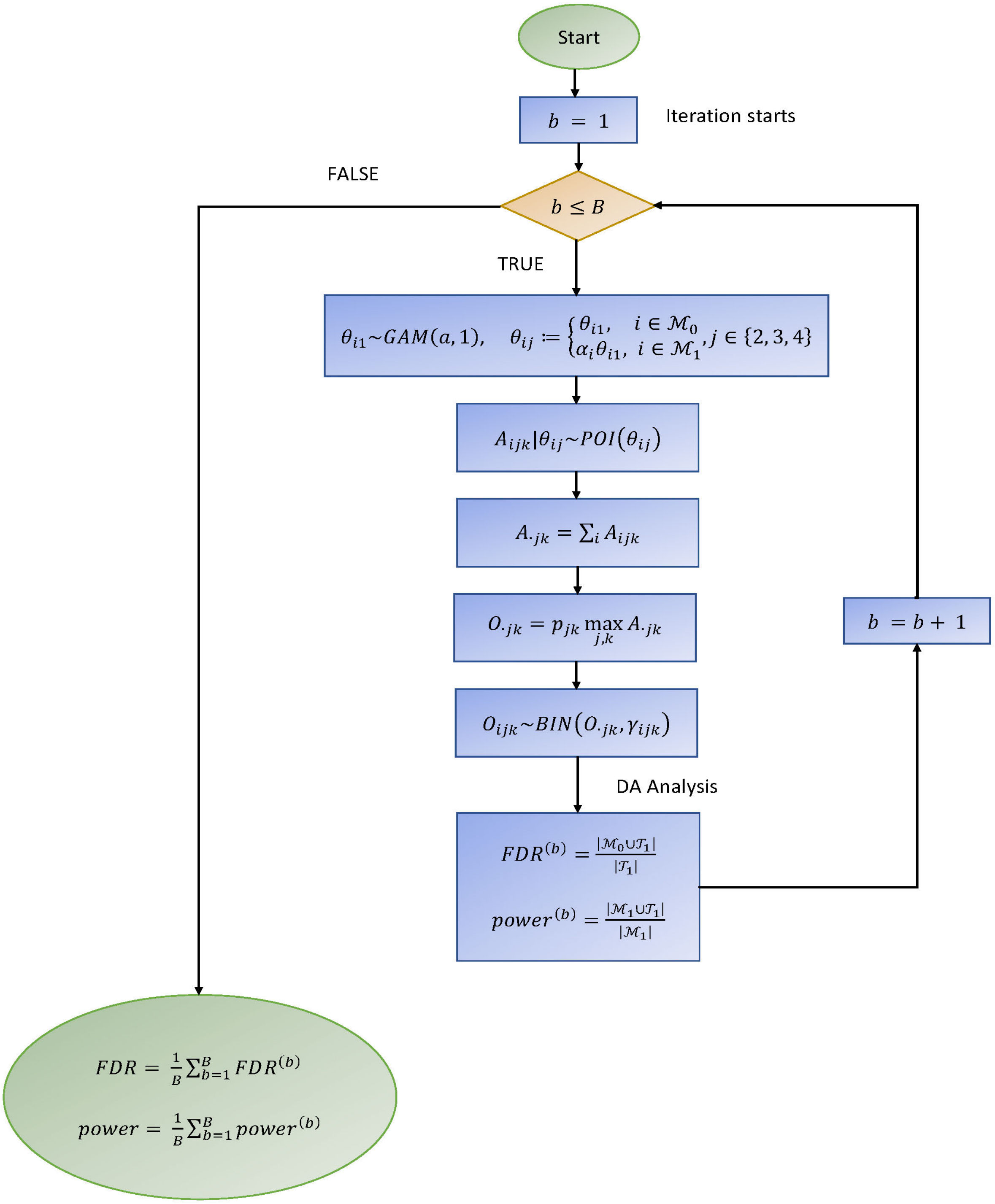

Supplementary Figure 14: Flowchart of simulation for FDR and power evaluation using Poisson-Gamma models: Multi-group comparison.

Supplementary Tables

Phylum	Log Fold Change	SE	CI.Lower	CI.Upper	S.Zero	P-value
			MA-US, infants			
Elusimicrobia	2.04	0.00	2.04	2.04	1.00	0.00
Spirochaetes	2.61	0.00	2.61	2.61	1.00	0.00
Cyanobacteria	2.60	0.32	1.68	3.52	0.00	0.00
Verrucomicrobia	-4.03	0.59	-5.75	-2.32	0.00	0.00
Fusobacteria	2.95	0.47	1.58	4.32	0.00	0.00
Tenericutes	-2.75	0.52	-4.26	-1.23	0.00	0.00
Lentisphaerae	0.51	0.29	-0.32	1.34	0.00	0.81
Actinobacteria	0.65	0.45	-0.66	1.95	0.00	1.00
Bacteroidetes	0.85	0.58	-0.84	2.53	0.00	1.00
Euryarchaeota	-0.12	0.24	-0.80	0.56	0.00	1.00
Firmicutes	-0.32	0.23	-0.99	0.35	0.00	1.00
Proteobacteria	-0.36	0.44	-1.62	0.90	0.00	1.00
TM7	0.04	0.25	-0.69	0.78	0.00	1.00
Synergistetes	0.00	0.00	0.00	0.00	1.00	1.00
			MA-US, adults			
Elusimicrobia	6.43	0.00	6.43	6.43	1.00	0.00
Spirochaetes	4.90	0.00	4.90	4.90	1.00	0.00
Synergistetes	-1.06	0.00	-1.06	-1.06	1.00	0.00
Cyanobacteria	6.26	0.96	3.47	9.04	0.00	0.00
Verrucomicrobia	-4.26	1.09	-7.44	-1.08	0.00	0.00
Lentisphaerae	3.55	1.09	0.39	6.72	0.00	0.01
Euryarchaeota	3.07	1.05	0.00	6.14	0.00	0.04
Actinobacteria	-1.91	0.76	-4.13	0.31	0.00	0.13
Bacteroidetes	0.11	0.70	-1.95	2.16	0.00	1.00
Firmicutes	-0.62	0.65	-2.53	1.28	0.00	1.00
Fusobacteria	0.87	0.92	-1.80	3.54	0.00	1.00
Proteobacteria	1.17	0.71	-0.90	3.25	0.00	1.00
Tenericutes	-0.08	0.67	-2.04	1.87	0.00	1.00
TM7	-0.75	0.66	-2.66	1.16	0.00	1.00
			VEN-US, infants			
Elusimicrobia	1.93	0.00	1.93	1.93	1.00	0.00
Spirochaetes	1.46	0.00	1.46	1.46	1.00	0.00
Cyanobacteria	4.17	0.54	2.61	5.73	0.00	0.00
Lentisphaerae	2.11	0.51	0.63	3.59	0.00	0.00
Fusobacteria	1.83	0.55	0.23	3.43	0.00	0.01
Bacteroidetes	1.49	0.61	-0.27	3.25	0.00	0.16
Actinobacteria	-0.24	0.68	-2.20	1.71	0.00	1.00
Euryarchaeota	0.39	0.39	-0.73	1.51	0.00	1.00
Firmicutes	-0.09	0.29	-0.92	0.73	0.00	1.00
Proteobacteria	-0.04	0.47	-1.41	1.33	0.00	1.00
Tenericutes	-0.13	0.60	-1.86	1.60	0.00	1.00
TM7	0.06	0.34	-0.92	1.03	0.00	1.00

Continued on next page

Supplementary Table 1 – *Continued from previous page*

**Phylum**	**Log Fold Change**	**SE**	**CI.Lower**	**CI.Upper**	**S.Zero**	**P-value**
Verrucomicrobia	-0.82	0.82	-3.19	1.56	0.00	1.00
Synergistetes	0.00	0.00	0.00	0.00	1.00	1.00
			VEN-US, adults			
Elusimicrobia	7.58	0.00	7.58	7.58	1.00	0.00
Spirochaetes	6.07	0.00	6.07	6.07	1.00	0.00
Firmicutes	-2.28	0.35	-3.31	-1.26	0.00	0.00
Cyanobacteria	3.88	0.64	2.03	5.73	0.00	0.00
Actinobacteria	-2.87	0.48	-4.27	-1.48	0.00	0.00
Verrucomicrobia	-4.28	0.78	-6.54	-2.02	0.00	0.00
TM7	-2.02	0.39	-3.17	-0.88	0.00	0.00
Bacteroidetes	-1.74	0.42	-2.97	-0.52	0.00	0.00
Synergistetes	-2.10	0.53	-3.66	-0.54	0.00	0.00
Lentisphaerae	2.69	0.69	0.67	4.71	0.00	0.00
Tenericutes	-1.10	0.42	-2.32	0.11	0.00	0.10
Euryarchaeota	0.74	0.79	-1.55	3.03	0.00	1.00
Fusobacteria	0.19	0.73	-1.95	2.32	0.00	1.00
Proteobacteria	-0.34	0.52	-1.85	1.18	0.00	1.00
			MA-VEN, infants			
Acidobacteria	0.33	0.00	0.33	0.33	1.00	0.00
Chloroflexi	0.26	0.00	0.26	0.26	1.00	0.00
Verrucomicrobia	-2.98	0.65	-4.87	-1.08	0.00	0.00
Tenericutes	-2.37	0.54	-3.95	-0.79	0.00	0.00
Spirochaetes	1.13	0.35	0.11	2.15	0.00	0.02
Lentisphaerae	-1.35	0.50	-2.82	0.12	0.00	0.09
Cyanobacteria	-1.31	0.53	-2.88	0.26	0.00	0.18
Fusobacteria	1.35	0.60	-0.40	3.09	0.00	0.31
Actinobacteria	1.13	0.60	-0.64	2.89	0.00	0.80
Bacteroidetes	-0.41	0.55	-2.03	1.21	0.00	1.00
Elusimicrobia	0.09	0.33	-0.89	1.07	0.00	1.00
Euryarchaeota	-0.27	0.33	-1.24	0.70	0.00	1.00
Firmicutes	0.01	0.22	-0.65	0.66	0.00	1.00
Proteobacteria	-0.11	0.39	-1.25	1.04	0.00	1.00
TM7	0.22	0.29	-0.64	1.08	0.00	1.00
Synergistetes	0.00	0.00	0.00	0.00	1.00	1.00
			MA-VEN, adults			
Synergistetes	-1.48	0.00	-1.48	-1.48	1.00	0.00
Verrucomicrobia	-1.65	0.76	-3.86	0.56	0.00	0.39
Elusimicrobia	1.67	0.83	-0.76	4.10	0.00	0.59
Actinobacteria	-0.67	0.52	-2.20	0.86	0.00	1.00
Bacteroidetes	0.20	0.46	-1.13	1.54	0.00	1.00
Cyanobacteria	0.70	0.68	-1.27	2.67	0.00	1.00
Euryarchaeota	0.66	0.69	-1.36	2.68	0.00	1.00
Firmicutes	0.01	0.41	-1.19	1.20	0.00	1.00
Fusobacteria	-0.98	0.71	-3.05	1.09	0.00	1.00
Lentisphaerae	-0.78	0.77	-3.01	1.45	0.00	1.00

Continued on next page

Supplementary Table 1 – *Continued from previous page*

**Phylum**	**Log Fold Change**	**SE**	**CI.Lower**	**CI.Upper**	**S.Zero**	**P-value**
Proteobacteria	-0.14	0.46	-1.47	1.19	0.00	1.00
Spirochaetes	1.65	1.03	-1.34	4.65	0.00	1.00
Tenericutes	-0.63	0.40	-1.79	0.53	0.00	1.00
TM7	-0.40	0.41	-1.59	0.79	0.00	1.00
Acidobacteria	0.00	0.00	0.00	0.00	1.00	1.00
Chloroflexi	0.00	0.00	0.00	0.00	1.00	1.00

Supplementary Table 1: Results of pairwise differential abundance analyses stratified by age: infants (at most 2 years), and adults (between 18 and 40) using the global gut microbiota data [10]

Differential abundance analyses were performed at the phylum level of the taxonomy. Effect size (log fold change), standard error (SE), Bonferroni adjusted 95% confidence intervals (CI), and p-value (two-sided; Bonferroni adjusted) are provided. Presence of structural zero (S.Zero) is denoted by 1 in the corresponding column and the taxon would be declared as differential abundant automatically (with zero SE and zero adjusted p-value).

Infants (age $\leq$ 2, n = 133)			
	**Malawi (n = 56)**	**The US (n = 49)**	**Venezuela (n = 28)**
**Age**			
Min	0.033	0.083	0.250
Max	2.0	2.0	2.0
Mean (SD)	0.99 (0.63)	0.55 (0.42)	1.1 (0.58)
**BMI**			
Min	11	14	14
Max	22	24	19
Mean (SD)	16 (1.9)	18 (3.4)	16 (1.4)
**Gender (%)**			
F	26 (46)	26 (53)	10 (36)
M	30 (54)	23 (47)	15 (54)
NA	0 (0)	0 (0)	3 (11)
**Breast-Fed (%)**			
Y	56 (100)	10 (20)	28 (100)
N	0 (0)	27 (55)	0 (0)
NA	0 (0)	12 (24)	0 (0)
**Adults (18 $\leq$ age $\leq$ 40, n = 83)**			
	**Malawi (n = 21)**	**The US (n = 41)**	**Venezuela (n = 21)**
**Age**			
Min	20	23	18
Max	38	40	40
Mean (SD)	27 (4.9)	29 (5.3)	29 (7.4)
**BMI**			
Min	20	18	21
Max	26	66	41
Mean (SD)	22 (2.0)	27 (11)	30 (5.2)
**Gender (%)**			
F	21 (100)	39 (95)	20 (95)
M	0 (0)	2 (5)	1 (5)
NA	0 (0)	0 (0)	0 (0)
**Breast-Fed (%)**			
Y	0 (0)	0 (0)	0 (0)
N	0 (0)	0 (0)	0 (0)
NA	21 (100)	41 (100)	21 (100)

Supplementary Table 2: Summary of demographic variables of the global gut microbiota data[10].

Infants (age $\leq$ 2)			
	**Log fold change**	**SE**	**P-value**
**MA - US**	1.2	0.63	0.063
**VEN - US**	1.6	0.67	0.018*
**MA - VEN**	-0.41	0.59	0.48
**Adults (18 $\leq$ age $\leq$ 40)**			
	**Log fold change**	**SE**	**P-value**
**MA - US**	0.73	0.96	0.45
**VEN - US**	0.54	0.55	0.33
**MA - VEN**	0.20	0.62	0.75

Supplementary Table 3: Pairwise tests using ANCOM-BC for the equality of mean log ratio of Bacteroidetes to Firmicutes between two populations.

Data are represented by effect size (log fold change), standard error (SE), and p-value (two-sided; Bonferroni adjusted) derived from the ANCOM-BC model. The differences are represented as Population X - Population Y. Thus, in the case of adult populations, the mean ratio of Bacteroidetes to Firmicutes in Malawi is $\exp(0.73) = 2.08$ times more than in the US. It is well-known that the ratio of Bacteroidetes to Firmicutes is inversely related to BMI (or obesity). According to the global gut microbiota data [10], the average BMI of US adult is larger than that of a Malawi adult (independent of gender). Similarly, the mean ratio of Bacteroidetes to Firmicutes in Venezuela adult population is $\exp(0.54) = 1.72$ times more than in the US.

# Taxa	Sample Size	Diff (%)	FDR	FDRSD	Power	PowerSD
10	20/30	25	0	0	0.96	0.14
10	50/50	25	0.0073	0.07	0.96	0.13
50	20/30	25	0.012	0.037	0.79	0.15
50	50/50	25	0.012	0.047	0.84	0.13

Supplementary Table 4: FDR and power of ANCOM-BC when the number of taxa is small.

Data are generated from Poisson-Gamma distributions. The variability in sampling fractions is set to be large and the proportion of differentially abundant taxa (Diff (%)) is set to be 25%. Two simulation scenarios are considered: small and unbalanced data ($n_1 = 20$, $n_2 = 30$), as well as large and balanced data ($n_1 = n_2 = 50$); number of simulations = 100. FDR and power from ANCOM-BC model (two-sided; Bonferroni adjusted) were evaluated at small number of taxa (10 or 50). As we increase the absolute abundance mimicking the OTU table aggregating to higher taxonomic levels (e.g. Phylum level), ANCOM-BC still manages to control the FDR in this condition.

# Taxa	Sample Size	Diff (%)	FDR	FDRSD	Power	PowerSD
1000	20/20/30/30	5	0.033	0.049	0.95	0.014
1000	20/20/30/30	15	0.030	0.056	0.89	0.018
1000	20/20/30/30	25	0.026	0.048	0.86	0.019
1000	50/50/50/50	5	0.024	0.040	0.97	0.012
1000	50/50/50/50	15	0.025	0.054	0.92	0.014
1000	50/50/50/50	25	0.031	0.057	0.90	0.015

Supplementary Table 5: FDR and power of ANCOM-BC for multi-group comparison.

Data are generated from Poisson-Gamma distributions. The variability in sampling fractions is set to be large, sample size is set to be either 20/20/30/30 or 50/50/50/50, and the proportion of differentially abundant taxa (Diff (%)) ranges from 5% to 25%; number of simulations = 100.. FDR and power from ANCOM-BC model (two-sided; Bonferroni adjusted) were evaluated in the presence of 5=4 group. ANCOM-BC successfully controls the FDR and maintains high power in all simulation scenarios.

Diff (%)	Bias^2 (EM)	Bias^2 (WLS)	Var (EM)	Var (WLS)	$1/(nm_0)$
5	5.30×10^{-4}	7.49×10^{-4}	1.85×10^{-5}	2.27×10^{-5}	2.11×10^{-5}
15	0.186	0.165	4.94×10^{-5}	7.56×10^{-5}	2.35×10^{-5}
25	0.0291	0.0449	1.92×10^{-5}	6.13×10^{-5}	2.67×10^{-5}

Supplementary Table 6: Bias and variance of $\hat{\delta}_{\text{EM}}$ and $\hat{\delta}_{\text{WLS}}$.

Bias (squared) and variance of $\hat{\delta}_{\text{EM}}$ and $\hat{\delta}_{\text{WLS}}$ with respect to δ were calculated under different simulation scenarios. Sample size is set to be 50/50, and the proportion of differentially abundant taxa (Diff (%)) ranges from 5% to 25%; number of simulations = 100. Both EM and WLS estimators have relatively small bias in estimating δ, and their variances are of the order of $1/(nm_0)$.

Method	Estimate of c_{jk}	Estimate of d_{jk}
ANCOM-BC	$\hat{c}_{jk}^{\text{ANCOM-BC}} = \exp(\hat{d}_{jk}^{\text{ANCOM-BC}})$	$\hat{d}_{jk}^{\text{ANCOM-BC}} = \begin{cases} \bar{y}_{\cdot rk} - \bar{y}_{\cdot r \cdot} & j = r \\ \bar{y}_{\cdot jk} - \bar{y}_{\cdot j \cdot} - \hat{\delta}_{rj} & j \neq r \end{cases}$, where r is the reference group.
CSS	$\hat{c}_{jk}^{\text{CSS}} = \exp(\hat{d}_{jk}^{\text{CSS}})$	$\hat{d}_{jk}^{\text{CSS}} = \log_2(\frac{s_{jk}^{\hat{l}} + 1}{N})$.
MED	$\hat{c}_{jk}^{\text{MED}} = \underset{i: O_i^R \neq 0}{\text{median}} \frac{O_{ijk}}{O_i^R}$	$\hat{d}_{jk}^{\text{MED}} = \log(\hat{c}_{jk}^{\text{MED}})$.
UQ	$\hat{c}_{jk}^{\text{UQ}} = \underset{i: O_{ijk} > 0}{\text{UQ}} (\frac{O_{ijk}}{O_{\cdot jk}})$, where UQ denotes the upper quartile.	$\hat{d}_{jk}^{\text{UQ}} = \log(\hat{c}_{jk}^{\text{UQ}})$.
TMM	$\log_2(\hat{c}_j^{\text{TMM}}) = \frac{\sum_{i \in G*} w_{ijk} M_{ijk}}{\sum_{i \in G*} w_{ijk}}$, where $M_{ijk} = \log_2(\frac{O_{ijk}/O_{\cdot jk}}{O_{ijr}/O_{\cdot jr}})$, $w_{ij} = \frac{O_{\cdot jk} - O_{ijk}}{O_{\cdot jk} O_{ijk}} + \frac{O_{\cdot jr} - O_{ijr}}{O_{\cdot jr} O_{ijr}}$. Refer to Robinson and Oshlack [11] for details.	$\hat{d}_{jk}^{\text{TMM}} = \log(\hat{c}_{jk}^{\text{TMM}})$.
Elib-UQ	$\hat{c}_{jk}^{\text{Elib-UQ}} = O_{\cdot jk} \hat{c}_{jk}^{\text{UQ}}$	$\hat{d}_{jk}^{\text{Elib-UQ}} = \log(\hat{c}_{jk}^{\text{Elib-UQ}})$.
Elib-TMM	$\hat{c}_{jk}^{\text{Elib-TMM}} = O_{\cdot jk} \hat{c}_{jk}^{\text{TMM}}$	$\hat{d}_{jk}^{\text{Elib-TMM}} = \log(\hat{c}_{jk}^{\text{Elib-TMM}})$.
TSS	$\hat{c}_{jk}^{\text{TSS}} = O_{\cdot jk}$	$\hat{d}_{jk}^{\text{TSS}} = \log(\hat{c}_{jk}^{\text{TSS}})$.

Supplementary Table 7: Summary of different estimators of sampling fractions.

Supplementary References

[1] Henry B Mann and Donald R Whitney. On a test of whether one of two random variables is stochastically larger than the other. *The annals of mathematical statistics*, pages 50–60, 1947.

[2] Michael I Love, Wolfgang Huber, and Simon Anders. Moderated estimation of fold change and dispersion for rna-seq data with deseq2. *Genome biology*, 15(12):550, 2014.

[3] Mark D Robinson, Davis J McCarthy, and Gordon K Smyth. edger: a bioconductor package for differential expression analysis of digital gene expression data. *Bioinformatics*, 26(1):139–140, 2010.

[4] Joseph N Paulson, O Colin Stine, Héctor Corrada Bravo, and Mihai Pop. Differential abundance analysis for microbial marker-gene surveys. *Nature methods*, 10(12):1200, 2013.

[5] Yunshun Chen, Davis McCarthy, Mark Robinson, and Gordon K Smyth. edger: differential expression analysis of digital gene expression data user's guide, 2014.

[6] Andrew D Fernandes, Jean M Macklaim, Thomas G Linn, Gregor Reid, and Gregory B Gloor. Anova-like differential expression (aldex) analysis for mixed population rna-seq. *PLoS One*, 8(7), 2013.

[7] Andrew D Fernandes, Jennifer Ns Reid, Jean M Macklaim, Thomas A McMurrough, David R Edgell, and Gregory B Gloor. Unifying the analysis of high-throughput sequencing datasets: characterizing rna-seq, 16s rrna gene sequencing and selective growth experiments by compositional data analysis. *Microbiome*, 2(1):15, 2014.

[8] Gregory B Gloor, Jean M Macklaim, Vera Pawlowsky-Glahn, and Juan J Egozcue. Microbiome datasets are compositional: and this is not optional. *Frontiers in microbiology*, 8:2224, 2017.

[9] J Gregory Caporaso, Christian L Lauber, William A Walters, Donna Berg-Lyons, Catherine A Lozupone, Peter J Turnbaugh, Noah Fierer, and Rob Knight. Global patterns of 16s rrna diversity at a depth of millions of sequences per sample. *Proceedings of the national academy of sciences*, 108(Supplement 1):4516–4522, 2011.

[10] Tanya Yatsunenko, Federico E Rey, Mark J Manary, Indi Trehan, Maria Gloria Dominguez-Bello, Monica Contreras, Magda Magris, Glida Hidalgo, Robert N Baldassano, Andrey P Anokhin, et al. Human gut microbiome viewed across age and geography. *nature*, 486(7402):222, 2012.

[11] Mark D Robinson and Alicia Oshlack. A scaling normalization method for differential expression analysis of rna-seq data. *Genome biology*, 11(3):R25, 2010.
