# Supplementary material for: Analysis of compositions of microbiomes with bias correction
Source: Nat Commun. 2020 Jul 14;11:3514. doi: 10.1038/s41467-020-17041-7 (PMC7360769; doi:10.1038/s41467-020-17041-7)
Supplement: Supplementary file 3 — Reporting Summary [file 41467_2020_17041_MOESM3_ESM.pdf]

nature research

Corresponding author(s): Shyamal Das Peddada

Last updated by author(s): Mar 12, 2020

Reporting Summary

Nature Research wishes to improve the reproducibility of the work that we publish. This form provides structure for consistency and transparency in reporting. For further information on Nature Research policies, see Authors & Referees and the Editorial Policy Checklist .

Statistics

For all statistical analyses, confirm that the following items are present in the figure legend, table legend, main text, or Methods section.

n/a	Confirmed	
☐	☒	The exact sample size (n) for each experimental group/condition, given as a discrete number and unit of measurement
☒	☐	A statement on whether measurements were taken from distinct samples or whether the same sample was measured repeatedly
☐	☒	The statistical test(s) used AND whether they are one- or two-sided<br>*Only common tests should be described solely by name; describe more complex techniques in the Methods section.*
☐	☒	A description of all covariates tested
☐	☒	A description of any assumptions or corrections, such as tests of normality and adjustment for multiple comparisons
☐	☒	A full description of the statistical parameters including central tendency (e.g. means) or other basic estimates (e.g. regression coefficient) AND variation (e.g. standard deviation) or associated estimates of uncertainty (e.g. confidence intervals)
☐	☒	For null hypothesis testing, the test statistic (e.g. F, t, r) with confidence intervals, effect sizes, degrees of freedom and P value noted<br>*Give P values as exact values whenever suitable.*
☒	☐	For Bayesian analysis, information on the choice of priors and Markov chain Monte Carlo settings
☒	☐	For hierarchical and complex designs, identification of the appropriate level for tests and full reporting of outcomes
☐	☒	Estimates of effect sizes (e.g. Cohen's d, Pearson's r), indicating how they were calculated

Our web collection on statistics for biologists contains articles on many of the points above.

Software and code

Policy information about availability of computer code

Data collection	Data were collected and processed using RStudio. R version 3.6.2 (2019-12-12); Platform: x86_64-apple-darwin15.6.0 (64-bit); Running under: macOS 10.15.4.
Data analysis	Analyses were performed using RStudio. R version 3.6.2 (2019-12-12); Platform: x86_64-apple-darwin15.6.0 (64-bit); Running under: macOS 10.15.4; All analyses can be found under https://github.com/FrederickHuangLin/ANCOM-BC.

For manuscripts utilizing custom algorithms or software that are central to the research but not yet described in published literature, software must be made available to editors/reviewers. We strongly encourage code deposition in a community repository (e.g. GitHub). See the Nature Research guidelines for submitting code & software for further information.

Data

Policy information about availability of data

All manuscripts must include a data availability statement. This statement should provide the following information, where applicable:
- Accession codes, unique identifiers, or web links for publicly available datasets
- A list of figures that have associated raw data
- A description of any restrictions on data availability

DNA sequences from the global gut mirobiota study can be found in MG-RAST server under search string "mgp401" for Illumina V4-16S rRNA.

Field-specific reporting

Please select the one below that is the best fit for your research. If you are not sure, read the appropriate sections before making your selection.

☒ Life sciences ☐ Behavioural & social sciences ☐ Ecological, evolutionary & environmental sciences

Life sciences study design

All studies must disclose on these points even when the disclosure is negative.

Sample size	No sample size calculation was performed. In global gut microbiota data. We specifically focused on comparing microbial compositions between infants (age <= 2, n = 133) and adults (18 <= age <= 40, n = 83). We believe these sample sizes are sufficient since in the simulation study, ANCOM-BC shows good FDR control and maintains decent power when the sample size is much smaller (n1 = 20, n2 = 30).
Data exclusions	Subjects whose age is between 2 to 18 (2 < age < 18) or greater than 40 (age > 40) were excluded in the global gut microbiota data as we would like to compare microbial compositions between infants and adults.
Replication	No experiments in study
Randomization	No experiments in study
Blinding	No experiments in study

Reporting for specific materials, systems and methods

We require information from authors about some types of materials, experimental systems and methods used in many studies. Here, indicate whether each material, system or method listed is relevant to your study. If you are not sure if a list item applies to your research, read the appropriate section before selecting a response.

Materials & experimental systems

n/a	Involved in the study	
☒	☐	Antibodies
☒	☐	Eukaryotic cell lines
☒	☐	Palaeontology
☒	☐	Animals and other organisms
☒	☐	Human research participants
☒	☐	Clinical data

Methods

n/a	Involved in the study	
☒	☐	ChIP-seq
☒	☐	Flow cytometry
☒	☐	MRI-based neuroimaging
